# Estimating binding properties of transcription factors from genome-wide binding profiles

**DOI:** 10.1093/nar/gku1269

**Published:** 2014-11-28

**Authors:** Nicolae Radu Zabet, Boris Adryan

**Affiliations:** 1 Cambridge Systems Biology Centre, University of Cambridge, Tennis Court Road, Cambridge CB2 1QR, UK; 2 Department of Genetics, University of Cambridge, Downing Street, Cambridge CB2 3EH, UK

## Abstract

The binding of transcription factors (TFs) is essential for gene expression. One important characteristic is the actual occupancy of a putative binding site in the genome. In this study, we propose an analytical model to predict genomic occupancy that incorporates the preferred target sequence of a TF in the form of a position weight matrix (PWM), DNA accessibility data (in the case of eukaryotes), the number of TF molecules expected to be bound specifically to the DNA and a parameter that modulates the specificity of the TF. Given actual occupancy data in the form of ChIP-seq profiles, we backwards inferred copy number and specificity for five *Drosophila* TFs during early embryonic development: Bicoid, Caudal, Giant, Hunchback and Kruppel. Our results suggest that these TFs display thousands of molecules that are specifically bound to the DNA and that whilst Bicoid and Caudal display a higher specificity, the other three TFs (Giant, Hunchback and Kruppel) display lower specificity in their binding (despite having PWMs with higher information content). This study gives further weight to earlier investigations into TF copy numbers that suggest a significant proportion of molecules are not bound specifically to the DNA.

## INTRODUCTION

Site-specific transcription factors (TFs) bind to the DNA and control the transcription rate of genes. Identifying the parameters influencing the interactions between TFs and DNA is essential in unveiling the gene regulatory program and better understanding the gene regulatory process. Significant insight has been gained by deriving the genome-wide binding profiles of TFs and, often, two complementary approaches have been combined to determine and analyse these genomic binding events, namely: (i) experimental determination of regions of genomic occupancy through chromatin immunoprecipitation experiments (ChIP-chip or ChIP-seq) (1) and (ii) computational inference of the very binding sites using various bioinformatics and biophysics approaches. In most cases, these computational approaches are based on scanning the DNA with a preferred DNA word, the so-called motif (often represented in the form of position weight matrix—PWM) (2). However, this approach discards effects from steric hindrance and competition on the DNA (3–5) or saturation of the binding sites due to high abundance of the TF (6–12).

An alternative to the bioinformatics approach is the statistical thermodynamics framework, which models the binding of TF molecules to DNA segments using the principles of physical chemistry (4,6–10,13–17). This approach considers both steric hindrance and the number of molecules that are bound to the DNA. Briefly, this framework computes the statistical weight for each possible configuration of the system, where a configuration represents the specific combination of locations on the DNA segment that are occupied by TF molecules. However, given the number of possible configurations, the computations of all statistical weights become challenging with increasing DNA segment size. To address this problem, we used several approximations within the statistical thermodynamics framework (10,18–20), which lead us to develop an analytical solution. This analytical model now allows us to compute binding profiles with the benefits of thermodynamics methods on a genomic scale (e.g. we computed the ChIP-seq profile of five TFs over 92 Mbp of DNA in less than 1 day using one CPU), instead of being restricted to a few loci compared to the classical approach as it was the case in some previous studies (4,13–17). This model takes as input four parameters: (i) a PWM, (ii) DNA accessibility data, (iii) the predicted or measured number of molecules that are specifically bound to the DNA and (iv) a factor that modulates the specificity of the TF (21). Whilst the first two parameters are often known—the PWM from *in vitro* experiments such as DNAse I footprinting, EMSA, SELEX or PBM (22) and the DNA accessibility data from genome-wide DNase I-seq experiments—the last two parameters are usually unknown and difficult to measure. Here, we show that the number of specifically bound molecules and the specificity of the TF can be computed by fitting the predictions of the model to experimentally determined binding profiles.

We applied our model on binding data of five TFs (Bicoid, Caudal, Giant, Hunchback and Kruppel) in the *Drosophila melanogaster* stage 5 embryo (23,24). Using the aforementioned rationale, we identified the number of DNA-bound molecules and the specificity for each of these TFs that fit the ChIP-seq signal with good accuracy. In particular, we estimate that the abundance of each of the TFs in the system is in the range of thousands of molecules that are specifically bound to the DNA per cell/nuclei. Finally, we also found that whilst Bicoid and Caudal display high specificity (being able to better discriminate between ‘good’ and ‘bad’ DNA words), Giant, Hunchback and Kruppel display lower specificity.

## MATERIALS AND METHODS

### Analytical model

In our previous work (11), we investigated the genomic occupancy of TFs using a comprehensive model that simulated the dynamics of TF molecules in the cell. We consider the ‘facilitated diffusion’ mechanism, which assumes that the molecules perform 3D diffusion in the cytoplasm/nucleoplasm and 1D random walk on the DNA (25–35). Our results showed that the genomic occupancy of TFs is mainly influenced by: (i) TF abundance and (ii) PWM information content (11). These results suggest that the statistical thermodynamics framework could accurately predict the genomic occupancy of TFs, although at a high computation cost. We addressed this issue, and in the Supplementary material we derive an analytical model based on statistical thermodynamics framework to compute the probability that a binding site is occupied as (see Supplementary Section S1)

(1) \begin{eqnarray*}
&&P^{{\rm bound}}_j\left(\lambda , w, N, a\right)\nonumber \\ &&=\frac{N\cdot a_j\cdot e^{\left(\frac{1}{\lambda }w_j\right)}}{N\cdot a_j\cdot e^{\left(\frac{1}{\lambda }w_j\right)} + L\cdot n\cdot \left\langle a_i e^{\left(\frac{1}{\lambda }w_i\right)}\right\rangle _i},
\end{eqnarray*}

where *N* is the number of molecules bound to the DNA, *a*_*j*_ represents the accessibility at site *j*, λ represents a scaling factor of the PWM score (8,21), *w*_*j*_ represents the PWM score at site *j*, *L* the length of the DNA and *n* is the ploidy level (the number of copies of the genome, e.g. for diploid genomes *n* = 2). When DNA accessibility data are discarded, then *a*_*j*_ = 1, ∀*j*.

### Data sets

**Figure 1.**
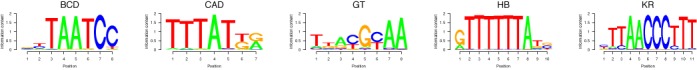
PWMs for the five TFs. The graph shows the sequence logos for the following TFs: (i) Bicoid, (ii) Caudal, (iii) Giant, (iv) Hunchback and (v) Kruppel as also used in (16). When computing the PWMs we used a pseudo-count of 1. The information content for the five motifs is: (i) *I*_BCD_ = 11.3, (ii) *I*_CAD_ = 10.7, (iii) *I*_GT_ = 8.7, (iv) *I*_HB_ = 15.6 and (v) *I*_KR_ = 17.3.

In Figure 1, we plot the sequence logos of the PWMs for the five TFs included in our analysis (Bicoid, Caudal, Giant, Hunchback and Kruppel); Berkeley *Drosophila* Transcription Network Project (bdtnp.lbl.gov) (16). To generate occupancy profiles we used a method originally introduced by (16), for which we selected a mean segment length of 200 bp, a standard deviation of 200 bp and the profile was smoothed over 250 bp; see also (11). First, we consider all the loci from (16), which are also listed in Supplementary Table S3.

In our analysis, we also consider DNA accessibility data derived from DNase-seq experiments in stage 5 *D. melanogaster* embryos. The raw data are from (36) and were used to compute the probability of accessible DNA; see (16) and Supplementary Equation (S9). In addition, we also used in our analysis the set of DNA accessible regions computed at a 5% false discovery rate (14.5 Mbp) (24) and represented accessible sites by *a* = 1 and inaccessible regions by *a* = 0; note that the dm3 release 5 coordinates of these regions were downloaded from ftp://hgdownload.cse.ucsc.edu/goldenPath/dm3/database/bdtnpDnaseAccS5.txt.gz. The *D. melanogaster* genome consists of the euchromatin (chromosomes chr2L, chr2R, chr3L, chr3R, chr4, chrX), heterochromatin (chr2LHet, chr2RHet, chr3LHet, chr3RHet, chrXHet, chrYHet), unmapped regions (ChrU and chrUextra) and mitochondrial genome (ChrM) (37). Only 5.6% of the DNase-seq reads from (36) map to heterochromatin or unmapped regions and only 3.1% of the total accessible DNA is in heterochromatin or on ChrU. The contribution of the heterochromatin and ChrU to our analysis is negligible and, thus, we considered in our analysis only the euchromatic genome (≈120 Mbp).

### Quantifying the differences between the analytical model and experimental data

To quantify the difference between our analytical model and the experimental data, we consider two measures, namely: (i) the Pearson correlation coefficient (*ρ*) and (ii) the normalized mean squared error over 1 kb (MSE).

## RESULTS

We applied our analytical model (derived in the Materials and Methods section and the Supplementary material) to investigate the ChIP-seq data set published in (23), which lead to a direct comparison to the method proposed in (16). This data set consists of ChIP-seq profiles determined in stage 5 *D. melanogaster* embryos for five TFs: (i) Bicoid (BCD), (ii) Caudal (CAD), (iii) Giant (GT), (iv) Hunchback (HB) and (v) Kruppel (KR).

Our analytical model requires four parameters: (i) the PWM for the factor under investigation, (ii) the DNA accessibility data over the locus that is analysed, (iii) the number of bound molecules (as can be inferred from genomic binding data) and (iv) the specificity of the TF for the DNA (through the λ factor); see Equation (1). We used the PWMs presented in Figure 1 and treated DNA either as ‘naked’ or used DNA accessibility from previously published work (16,24,36).

The number of bound molecules and TF specificity λ are usually unknown. Here, we estimate these parameters by identifying the values that produce the best fit with the experimentally measured profile (7,8,10,17). First, we converted the binding probability determined by Equation (1) into an occupancy profile (an artificial ChIP-seq signal; using Supplementary Equation (S8)) and then we generate the ChIP-like *in silico* profiles using a method described by (16), (selecting a mean segment length of 200 bp and a standard deviation of 200 bp and then smoothing the profile over 250 bp); the R implementation of this method is described in (11).

**Figure 2.**
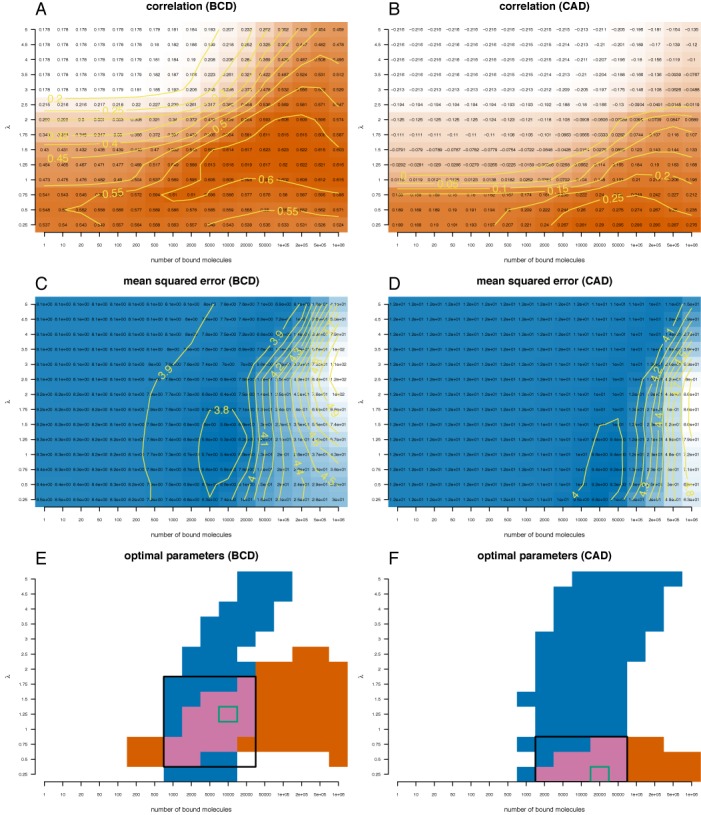
Quantifying the distances between Bicoid and Caudal ChIP-seq profiles and the profiles derived with the analytical model. We plotted heatmaps for the correlation (**A**) and (**B**) and mean squared error (**C**) and (**D**) between the analytical model and the ChIP-seq profile of Bicoid (A, C) and Caudal (B, D). We computed these values for different sets of parameters: *N* ∈ [1, 10^6^] and λ ∈ [0.25, 5]. We considered only the sites that have a PWM score higher than 70% of the difference between the lowest and the highest score. (A, B) Orange colour indicates high correlation between the analytical model and the ChIP-seq profile, whilst white colour low correlation. (C, D) Blue colour indicates low mean squared error between the analytical model and the ChIP-seq profile, whilst white colour high mean squared error. (**E, F**) We plotted the regions where the mean square error is in the lower 12% of the range of values (blue) and the correlation is the higher 12% of the range of values (orange). With green rectangle we marked the optimal set of parameters in terms of mean squared error and with a black rectangle the intersection of the parameters for which the two regions intersect.

Figure 2 plots the heatmap of the correlation and mean squared error for Bicoid and Caudal when the number of bound molecules and λ factor are varied. We performed a grid search to identify the sets of parameters (TF abundance and specificity) which maximizes the correlation and minimizes the mean squared error. Our results show that the set of parameters that minimizes the mean squared error leads to only a negligible reduction in the correlation compared to its maximum value. In contrast, the parameters that maximize the correlation lead to a strong increase in the mean squared error compared to its minimum. This means that changes in the λ factor and the TF abundance have a stronger effect on the mean squared error than on correlation. In a similar way, we determined the set of parameters that minimize the mean squared error and maximize the correlation for the rest of TFs (Giant, Hunchback and Kruppel) by analysing the data on the heatmaps in Supplementary Figure S4. Table 1 lists the optimal set of parameters for all five TFs.

**Table 1.** Set of parameters that minimizes the difference between the ChIP-seq profile and the analytical model

	*N*	λ	MSE	*ρ*
BCD	10000	1.25	5.29 (5.29)	0.62 (0.62)
CAD	20000	0.25	8.82 (8.82)	0.29 (0.30)
GT	1e+05	5.00	0.96 (0.96)	0.12 (0.31)
HB	5000	2.00	2.93 (2.93)	0.33 (0.38)
KR	20000	4.00	6.70 (6.70)	0.39 (0.41)

We also listed the values for the mean squared error (MSE) and correlation (*ρ*). The values in the parentheses represent the minimum mean squared error and the maximum correlation. We considered only the sites that have a PWM score higher than 70% of the distance between the lowest and the highest score.

### DNA accessibility improves the model predictions

One of the main results of (10,16,17) is that DNA accessibility data improve the computational prediction when estimating ChIP-seq profiles with PWMs. To investigate this result, we also consider DNA accessibility regions from stage 5 *D. melanogaster* embryos computed with a 5% false discovery rate (24) and represented accessible sites by *a* = 1 (14.5 Mbp) and inaccessible sites by *a* = 0.

**Figure 3.**
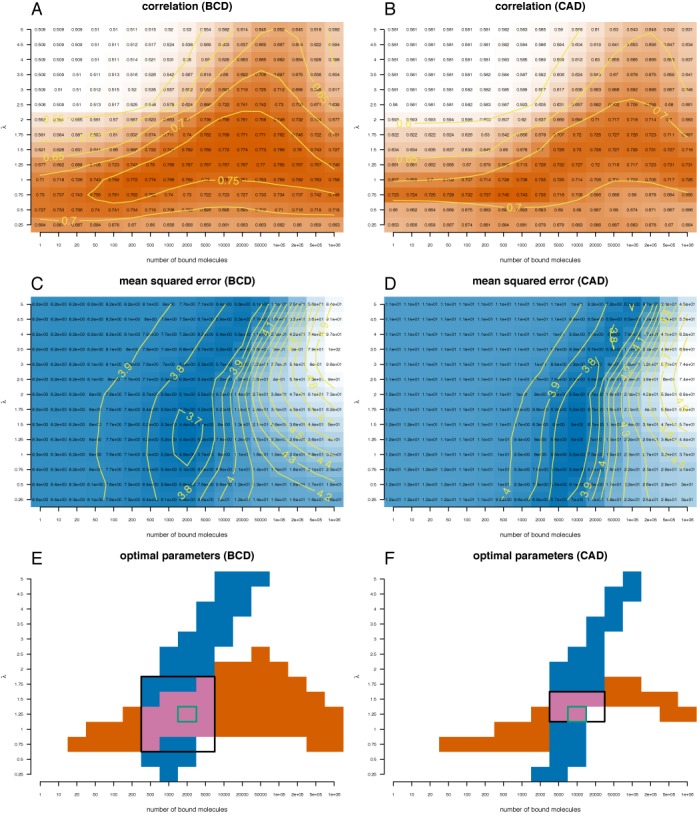
Quantifying the distances between Bicoid and Caudal ChIP-seq profiles and the profiles derived with the analytical model that includes DNA accessibility data. This is the same as Figure 2, except that we included binary DNA accessibility data in the analytical model.

Using the same approach as in the case of all DNA being accessible, we plot the heatmaps for correlation and mean squared error for Bicoid, Caudal, Giant, Hunchback and Kruppel and then we performed a grid search to identify the combination of parameters that minimize the differences between the ChIP-seq profiles and the profiles predicted by Equations (1) and Supplementary Equation (S8); see Figure 3 and Supplementary Figure S5.

**Figure 4.**
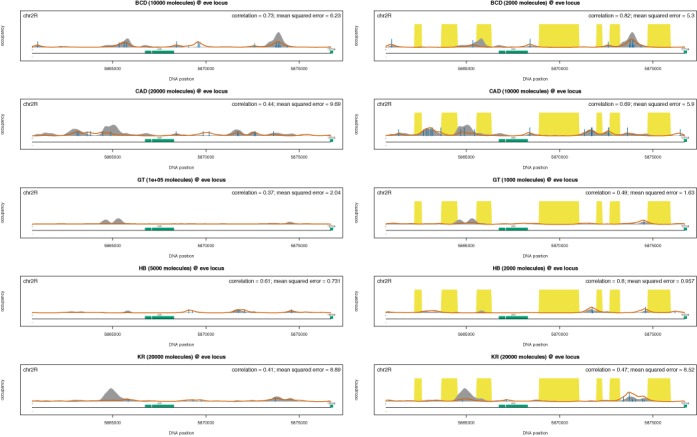
Binding profiles at eve locus. The grey shading represents a ChIP-seq profile, the red line represents the prediction of the analytical model, the yellow shading represents the inaccessible DNA and the vertical blue lines represent the percentage of occupancy of the site (we only displayed sites with an occupancy higher than 5%). We plotted the profiles for the five TFs: (i) Bicoid, (ii) Caudal, (iii) Giant, (iv) Hunchback and (v) Kruppel. (Left) The analytical model assumed a naked DNA (the entire genome is accessible) and used the set of parameters listed in Table 1. (Right) The analytical model included DNA accessibility data from (16,24) and used the set of parameters listed in Table 2.

Table 2 lists the optimal set of parameters for the five TFs in the case of DNA accessibility. Again, one can see that selecting the set of parameters that minimize the mean squared error leads to only negligible reduction in the correlation. Our results confirm that DNA accessibility data improve the model prediction (increase the correlation and reduce the mean squared error) and, thus, support the finding that DNA accessibility is a significant factor that drives the genomic occupancy of TFs; compare Table 1 to Table 2 and see Figure 4. Overall the correlation between our model predictions and the ChIP-seq data sets is similar to the one found in (16).

**Table 2.** Set of parameters that minimizes the difference between the ChIP-seq profile and the analytical model which includes DNA accessibility

	*N*	λ	MSE	*ρ*
BCD	2000	1.25	4.40 (4.40)	0.77 (0.77)
CAD	10000	1.25	5.03 (5.03)	0.73 (0.75)
GT	1000	1.00	0.85 (0.85)	0.55 (0.57)
HB	2000	3.00	2.38 (2.38)	0.66 (0.66)
KR	20000	5.00	4.77 (4.77)	0.68 (0.69)

The accessibility of any site can be either 0 or 1 depending on whether the site is accessible or not. We also listed the values for the mean squared error (MSE) and correlation (*ρ*). The values in the parentheses represent the minimum mean squared error and the maximum correlation. We considered only the sites that have a PWM score higher than 70% of the distance between the lowest and the highest score.

Interestingly, Figure 3E and F shows that there is a high correlation between our model and the ChIP-seq data for a wide range of values for the number of DNA-bound molecules (orange area). However, the range of values of λ that results in high correlation is much smaller. In contrast, the mean squared error is reduced only for a small interval of TF abundances, but it is optimal for a wide range of values for λ (blue area). This result suggests that the correlation between the model and the ChIP-seq profile cannot be used to estimate TF abundance (as previously done), but can accurately estimate the range of values for λ. In contrast, the mean squared error can be used to estimate the number of bound molecules, but cannot be used to estimate the λ factor. Thus, to get a better estimate for both the number of DNA-bound molecules and λ one needs to consider both correlation and mean squared error and identify the range of parameters where both measures are optimal.

It is also worthwhile noting that, for all TFs, the number of bound molecules that best fit the data is in the range of thousands of molecules bound to the genome (and is on average five times lower than the case of naked DNA). In addition, we identified that, for Hunchback and Kruppel, the values of λ that optimizes the analytical model are significantly higher than 1, which means that although the motifs of these two TFs have high information content (see Figure 1), the two TFs have low specificity and cannot distinguish well between different DNA words (22). In contrast, we found that Bicoid, Caudal and Giant display values of λ around 1, which indicates that the specificities of these TFs are equal to their information content, as defined by Stormo and Zhao (22).

### Additional factors that influence the binding profiles

Despite the generally accurate predictions of the model, there are locations on the genome where the model fails to predict the ChIP-seq profile. In the Supplementary material, we plotted the estimates of the binding profiles at all 21 loci using the optimal set of parameters (from Table 2); see Supplementary Figures S6–S10. Next, we systematically investigated several assumptions in our model that could account for the differences between the ChIP-seq data set and the model-predicted profiles.

#### Range of PWM scores included in the model

For our analysis we only considered predicted binding sites that display a PWM score which is higher than 70% of the difference between the strongest and the weakest site (∀*j*, where *w*_*j*_ ≥ 0.7 × [max _*i*_(*w*_*i*_) − min _*i*_(*w*_*i*_)]). To understand why we discard non-specific sites, it is important to remember that conventional ChIP experiments display a population average over millions of cells/nuclei. Whilst specific sites will be occupied in the majority of the cells (nuclei), a particular non-specific site will be occupied in a few cells, because there are many similar low affinity sites in the genome (38). This means that ChIP data reflect binding at the specific sites and that low affinity sites can be discarded. To test whether our threshold selection affected the results, we also considered the case of a lower threshold of 30%.

We found that weaker binding sites do not affect our model estimate for the profile for TFs that have low values of λ (Bicoid, Caudal and Giant); see Supplementary Table S4 and Supplementary Figure S11. However, for TFs with higher values of λ (Hunchback and Kruppel), weaker binding sites affect the binding profiles, but leading only to a negligible reduction in the quality of the profile generated by our model; see Supplementary Figure S12. Including lower affinity binding into our model also leads to a similar set of parameters (TF abundance and λ) that optimize the fit for four TFs (Bicoid, Caudal, Giant and Hunchback), thus, indicating that our estimates are robust. For Kruppel, when including lower affinity binding sites, our method estimates a lower TF abundance and λ. However, λ remains significantly higher than 1 and the quality of the fit is slightly worse that in the case of including only sites that have a PWM score higher than 70% of the difference between the lowest and the highest score; see Supplementary Table S4.

#### DNA accessibility data

Some DNA loci are marked as being ‘inaccessible’ in the DNA accessibility data, but, at the same time, display binding of TFs in ChIP experiments; see Supplementary Figures S6–S10. This suggests that regions with an intermediary level of DNA accessibility could have been marked as inaccessible despite allowing binding of TFs. To investigate this aspect, we also considered the case of different levels of DNA accessibility (the data are represented by continuous values between 0 and 1) and we converted the read density in probability of a site being accessible by using the approach described in (16); see Supplementary Equation (S9). We found that, when using non-binary accessibility data, the difference between the predictions of our model and the ChIP-seq data is similar to the case of using binary DNA accessibility data; see Supplementary Table S5 and Supplementary Figures S13 and S14.

#### Position weight matrices

Finally, we investigated whether the choice of PWMs affected our results by performing the analysis using alternative PWMs from the JASPAR database (39); see Supplementary Figure S15. One should note that whilst the motifs of Bicoid and Caudal are similar in both BDTNP (16) and JASPAR (39), the motif for Giant has a higher information content and the motifs for Hunchback and Kruppel have a lower information content in JASPAR (39) compared to BDTNP (16). Our results show that, by using a different set of PWMs, we obtained slightly worse values for correlation and mean squared error compared to the case of using the PWMs from BDTNP (16); see Supplementary Figures S16 and S17 and Supplementary Table S6. It remains to be investigated if this could be a generalized approach to determine the quality of PWMs from different sources.

Interestingly, we found that the values of λ that optimize the model for Giant, Hunchback and Kruppel differ significantly between the case of the PWMs from BDTNP (16) and the PWMs from JASPAR (39). This suggests that independent of the actual PWMs, the three TFs display similar specificity. Since three TFs have different PWMs, we also investigated the binding profiles at the 21 loci and we found that, in certain cases, the use of the PWMs from the JASPAR database leads to differences in the predicted profile; see Supplementary Figure S18–S20. For example, for Kruppel, we observed a slightly better estimation of the binding profile at *D*, *H*, *Kr*, *cad*, *ftz*, *gt*, *hkb*, *os* and *slp* loci and a slightly worse estimate of the profile at *cnc*, *croc*, *kni*, *opa*, *prd*, *run* and *tll* loci compared to the case when the PWMs from BDTNP (16) were used; compare Supplementary Figure S10 to Supplementary Figure S20.

### Genome-wide analysis of TF binding

**Figure 5.**
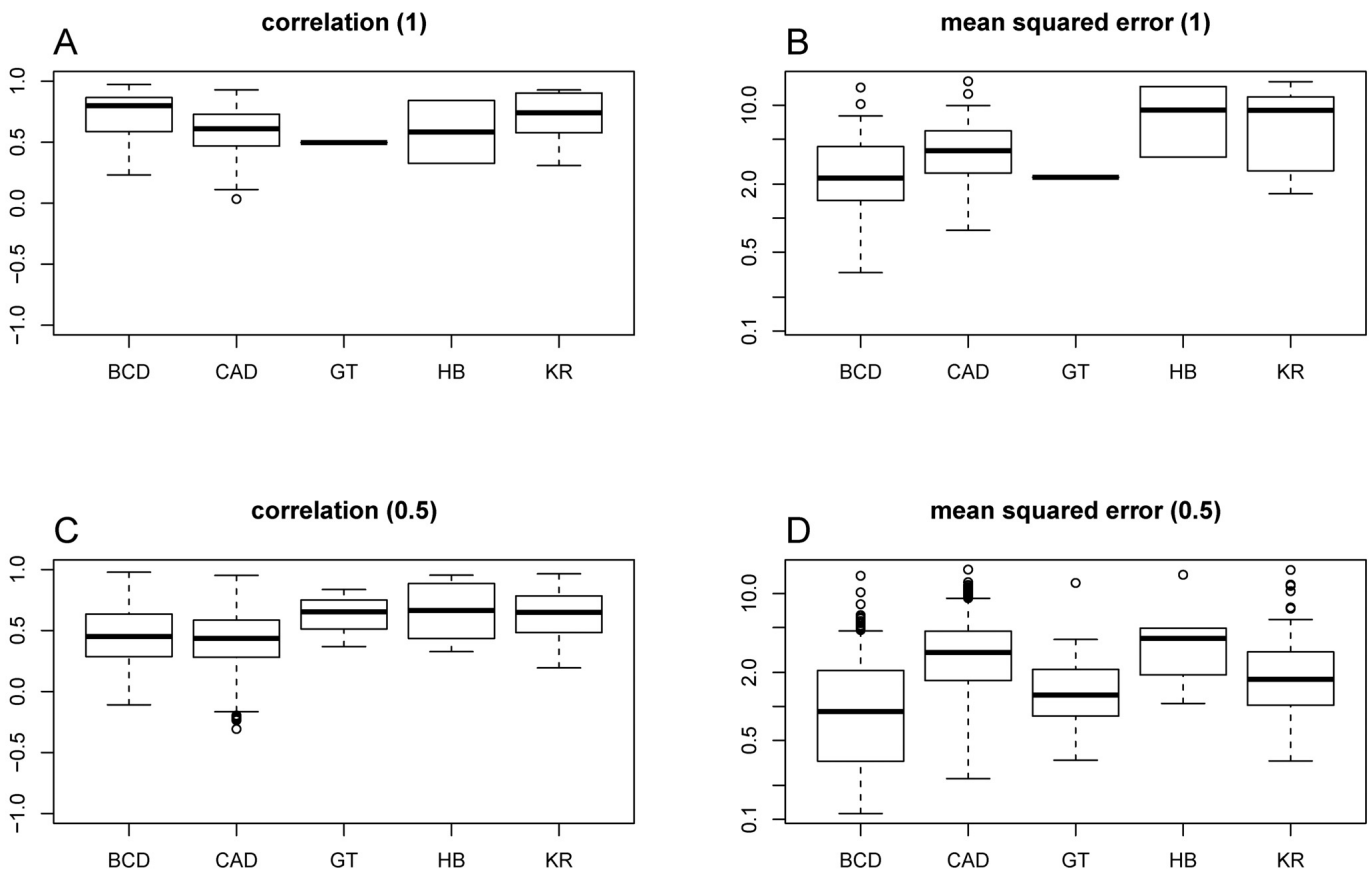
Genome-wide quality of the fit. The boxplots represent the (**A, C**) correlation and (**B, D**) mean squared error between the ChIP-seq data sets and the analytically estimated profiles. We partitioned the genome in 20*-*kb regions and we kept only the regions that had at least one DNA accessible site (4599 regions). Next for each ChIP-seq data set we selected the regions where the mean ChIP-seq signal is higher than a proportion of the background (see Supplementary Table S7). In (A, B), we selected the regions with a mean ChIP-seq signal higher than the background (>*B*). In (C, D), we selected the regions with a mean ChIP-seq signal higher than half the background (>0.5 · *B*). The numbers of DNA regions that display a mean ChIP-seq signal higher than the thresholds are listed in Supplementary Table S8. In all subgraphs we used the set of parameters from Table 2.

One advantage of our analytical model is that it can be used to predict the binding profiles genome-wide and, thus, we extended the analysis from the original 21 loci to the entire genome. We partitioned the genome in 20-kb regions, from which we removed regions that did not have any accessible site. For each ChIP-seq profile, we then selected the regions that display a ChIP-seq signal higher than the genome-wide background. We found that the quality of our model's predictions varies widely; see Figure 5A and B. In particular, there are regions where the correlation between our model predictions and the ChIP-seq profile is high, but at the same time regions where this correlation between our model's prediction and the ChIP-seq profile is low.

Kaplan *et al.* (16) found that, at loci with low binding (low ChIP-seq signal), the correlation between the statistical thermodynamics model and the ChIP-seq profile was low. To test whether this is valid genome-wide, we also analysed regions where the mean signal is higher than half of the genome-wide background (leading in an increase in the number of investigated loci). Our results confirm that there is a decrease in the mean correlation when including regions with lower ChIP-seq signal; see Figure 5C. We also perform a Kolmogorov–Smirnov test that showed that in the case of Bicoid and Caudal this difference is statistically significant; see Supplementary Figure S21. This also means that, at least for regions with strong binding, the model predictions are highly correlated with the ChIP-seq profile as previously found (16); see Figure 5. Nevertheless, for regions with low binding, in addition to the reduction in the correlation we also observed a decrease in the mean squared error, which is statistically significant in the case of Bicoid, Caudal and Kruppel; see Supplementary Figure S21. Note that for Giant and Hunchback the difference is not statistically significant due to the small number of loci included in the analysis; see Supplementary Table S8. This indicates that our model is able to correctly capture the low signal in those regions, but there is little or no correlation to the actual ChIP-seq signal. One explanation for this result is that, in those regions, there is little or no binding and what the ChIP-seq method recovers might be considered technical noise.

### Nucleus-specific binding predictions

**Figure 6.**
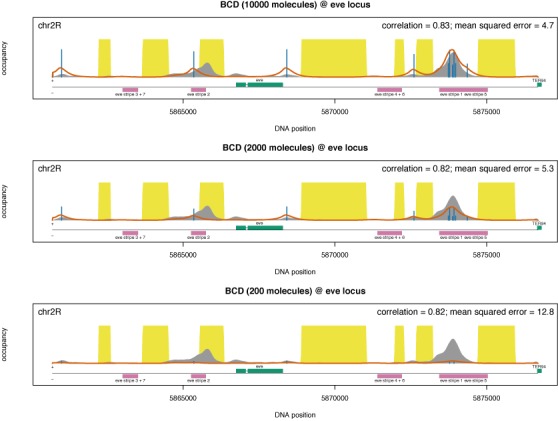
Bicoid binding profile at eve locus for various abundances. The grey shading represents the embryo-wide ChIP-seq profile of Bicoid, the red line represents the prediction of the analytical model, the yellow shading represents the inaccessible DNA and the vertical blue lines represent the percentage of occupancy of the site (we only displayed sites with an occupancy higher than 5%). We consider three cases (i) *N*_BCD_ = 10 000 molecules (anterior pole), (ii) *N*_BCD_ = 2000 molecules (stripe 2 region) and (iii) *N*_BCD_ = 200 molecules (posterior pole). We also assume a factor λ_BCD_ = 1.25. The magenta rectangles mark the enhancers for the stripe formation; from left to right these are: (i) eve stripe 3+7, (ii) eve stripe 2, (iii) eve stripe 4+6, (iv) eve stripe 1 and (v) eve stripe 5.

Using our model we can investigate the binding profiles at various locations for which ChIP-seq data are not available. Whilst the ChIP-seq profiles were generated for the entire embryo (and, thus, we are assuming average TF abundance over the pool of cells in an embryo), there is still no indication how these profiles look at specific locations along the anterior–posterior axis of the animal. This is important because, along the embryonic axis, the TF abundance can vary significantly; see (16,40–46). First, we generated the Bicoid binding profile in nuclei that are positioned at 40% of egg-length along the A-P axis from the anterior pole (stripe 2) by assuming that there are 2000 molecules of Bicoid in this region. In particular, we approximated at 40% of egg-length along the A-P axis the number of specifically bound molecules is similar to the one computed for the embryo-wide ChIP-seq. Figure 6 shows how this binding profile looks and confirms that Bicoid binds to the ‘eve stripe 2’ enhancer (chr2R:5865267-5865750), as opposed to the case of the posterior pole (with much lower concentration), where Bicoid does not bind to this enhancer. Note that we approximated that, at the posterior pole, the amount of Bicoid is 10 times lower than at the ‘eve stripe 2’ enhancer (41).

Next, we generated the Bicoid binding profiles at the anterior region of the embryo assuming that, in this region, the number of specifically bound Bicoid molecules is approximately five times higher than at the ‘eve stripe 2’ enhancer ((41) approximated that at the anterior pole there are approximately four times more Bicoid molecules than at the ‘eve stripe 2’ enhancer). Figure 6 shows that, at the anterior pole, there is significantly more binding of Bicoid to the ‘eve stripe 2’ enhancer, which raises the question of why there is no eve expression in that region. Initially, it was assumed that Bicoid acts only as an activator for the ‘eve stripe 2’ enhancer (40,47). However, a recent study (12) proposed that Bicoid has a dual role as both activator and repressor and this is controlled by its abundance, i.e. for low and medium abundances Bicoid activates ‘eve’, whilst for high abundance it will repress it. Our results support (without providing a mechanistic explanation) that Bicoid cannot be an activator for ‘eve’ at high abundances, because this would mean that ‘eve’ should be expressed at the anterior regions of the embryo, which contradicts the experimental observations (40,47) (assuming that expression equals occupancy).

## DISCUSSION

Gene regulation plays a significant role in cellular response to developmental, physiological or environmental signals. To better understand these processes, there is a need to move from genetic interaction models of gene regulation to more fine-grained models that include the regulatory sequence (48). In this manuscript, we proposed an analytical model that is able to compute genome-wide binding profiles of TFs, as opposed to more detailed computational models for the statistical thermodynamics framework that are limited to smaller DNA loci. Our model recapitulates the main driving forces in determining the genome-wide occupancy of a TF, namely: (i) the PWM, (ii) DNA accessibility, (iii) the number of TF molecules that are bound to the DNA and (iv) the specificity of the TF (how well it discriminates between ‘good’ and ‘bad’ sequences) through the λ factor (21). Frequently, we have data for the first two and we aim to determine the last two by fitting the profile predicted by the model to the profile generated experimentally (through ChIP-seq) (8,10,14,16,17).

### Abundance of bound TF

Previous studies quantified the accuracy of predictions by determining the set of parameters (usually the number of TF molecules that are bound to the genome) that maximize the correlation between the computationally and experimentally generated profiles (10,17). Here, we considered the ChIP-seq data set for five TFs in the *D. melanogaster* embryo during early development and computed the Pearson correlation coefficient between the analytical and experimental profiles. In addition, we also computed the mean squared error between the computational and the ChIP-seq profiles. Our results confirm that DNA accessibility improves the predictions of the model (10,16,17) and show that the set of parameters that maximize the correlation also leads to high mean squared errors, whilst the set of parameters that minimize the mean squared errors leads to only a small decrease in the correlation (compared to the maximum). Furthermore, it seems that the correlation is less sensitive to changes in the TF abundance, but highly sensitive for changes in λ; e.g. the orange regions in Figure 3E and F are stretched horizontally. In contrast, the mean squared error is highly sensitive to changes in the number of TF molecules, but less sensitive to changes in λ; e.g. the blue regions in Figure 3E and F are stretched vertically. Together, this suggests that correlation could be used to estimate the λ factor and mean squared error to infer the amount of bound TF. Thus, our results indicate that when aiming to maximize the correlation, previous studies (8,10,14,16,17) potentially overestimated the number of TF molecules that are bound to the DNA.

For example, some work suggests the number of Bicoid molecules in the early fly embryo to be around 1.5 × 10^8^  molecules (44). Bicoid displays a gradient along the anterior–posterior axis of the embryo with most of the TFs located in the anterior pole. Assuming that the ChIP-seq signal mainly comes from the nuclei with high abundance, that ≈30% of the nuclei display high Bicoid abundance and that there are 6000 nuclei in the blastoderm embryo (43), one can compute the average number of Bicoid molecules per nucleus to be ≈80 000. Some of the molecules will be localized to the nucleus whilst others will diffuse in the cytoplasm. Gregor *et al.* (49) estimated that only 40% of Bicoid is nuclear, which means that the nuclear abundance of Bicoid is ≈30 000  molecules. In a subsequent study, the same group as in (44) proposed a slightly lower abundance of Bicoid in the *D. melanogaster* embryo, namely 4.5 × 10^7^  molecules (46). Following the same logic, we computed that there are 10 000 molecules of Bicoid per nucleus and this indicates that the Bicoid nuclear abundance can be estimated to be between 10 000 and 30 000 molecules. In accordance with these estimates, Abu-Arish *et al.* (42) estimated the Bicoid nuclear abundance as 140 nM, which is equivalent to 12 000 molecules per nucleus when using the estimate of nuclear volume from (41). There is a significant difference between the number of molecules that our model predicts (≈1000–5000 for all cases when DNA accessibility was included) and the number of molecules estimated in these experimental studies (10 000–30 000). However, our model estimates the number of molecules that are actually bound to the DNA, whilst other previous studies of (42,44,46) are based on the entire nuclear abundance of Bicoid.

Furthermore, TFs can bind specifically to high affinity site, but also non-specifically anywhere on the genome where they potentially perform 1D sliding on the DNA (25,26,32,33,50,51). Nevertheless, experimental studies have shown that ChIP only recovers specific binding of the TF to the DNA (52–55). ChIP is a population average measurement, which means that what it reports is the proportion of cells in which a specific locus was bound. Due to their high affinity, specific sites will be occupied in the majority of the cells (nuclei). In contrast, individual non-specific sites will be occupied in a few cells, because there are many more similar low affinity sites in the genome (38). Thus, ChIP data describe binding at the specific sites and this means that, when we estimate the number of bound TF molecules, in fact we estimate the number of specifically bound TF molecules.

In Supplementary Table S9 and Supplementary Figure S22, we summarized the results of a series of studies (note that this is not an exhaustive list) of the estimated percentage of specifically bound TFs (50,56–62). These studies were performed in different mammalian cell lines (HeLa, 3134, H1299, MCF-7, U87, ES, NIH 3T3), using different techniques (Fluorescence Recovery After Photobleaching—FRAP, Fluorescence Correlation Spectroscopy—FCS, Single Molecule Tracking—SMT and Reflected Light-Sheet Microscope—RLSM) and in different conditions. The results indicate that the percentage of specifically bound TF ranges between 2.5 and 99.7% with a median of ≈20%.

In the case of Bicoid, if only 20% of the TF is specifically bound and there are molecules between 10 000 and 30 000 in the nucleus, then the amount of specifically bound TF is between 2000 and 6000 molecules. These values are similar to the values that we calculate (≈1000–5000 molecules) assuming different models for Bicoid (binary and continuous DNA accessibility data, including weak binding sites or using a different PWM). Furthermore, in (63), the authors proposed a lower limit for the nuclear abundance of the five TFs by analysing the FlyEx database (64). Their values are much lower than what we estimate in the case of Bicoid. This can be explained as the authors in (63) removed the highest 10% measurements when computing the averages. However, the nuclear abundances proposed in (63) can be used to estimate the abundance in the nucleus for Caudal, Giant, Hunchback and Kruppel relative to Bicoid. We used this strategy to estimate the nuclear abundance of these four TFs (measured in the number of molecules) and then we estimated the percentage of specifically bound TFs (based on the estimations of our analytical model and the nuclear abundance of TFs); see Supplementary Table S10. Our method supports an extensive body of literature that only a relatively small percentage (30% or less) of the molecules of TFs are bound specifically to the genome.

Previous studies suggested that the TF abundance in eukaryotic systems can be high; e.g. (65) estimated that there are between 10^4^ and 3 × 10^5^  molecules per TF, whilst the same author later estimated that the median of TFs abundances in a mouse NIH 3T3 cell line is 7.1 × 10^4^  molecules (66). A different group estimated that there are between 250 and 3 × 10^5^  molecules for each TF in mouse 3T3-L1 cells. Assuming that less than 30% of these TF molecules are bound specifically to the genome, we estimate that the median number of TF molecules that are specifically bound is less than 21 000 molecules.

Since only specifically bound TFs seem to influence the transcription process (61), it is more important to know the exact amount of specifically bound TF, rather than the entire concentration in the nucleus (7–9,67). Thus, the range of parameters found by our study will have a higher impact for further studies that model these biological systems, compared to other work that estimates the nuclear concentration of TFs. It is worthwhile to mention that the estimate for proportion of non-specifically bound TFs is in the same range with the proportion of specifically bound TFs (38,57), which suggests that the amount of TF bound to the genome would be in similar ranges (2000–40 000 molecules).

It is worthwhile to note that the accuracy of our method to estimate TF abundance is limited by the ChIP methodology to fully recover the quantitative aspects of TF binding. For example, the *in silico* ChIP-seq profiles of lacI in (11) seem to be similar for lacI abundances between 1 and 1000 molecules, which suggests that our method will not be able to correctly estimate abundances lower than 1000 molecules. Thus, our method will perform best for cases where differences in TF abundance lead to strong differences in the ChIP profiles.

### The specificity of TFs

Our model also predicted that TFs can display higher or lower specificity beyond the information content of the binding motif, through the coefficient that modulates the discrimination energy between strong and weak binding sites (λ). Our results show that the difference between the binding energy of strong and weak sites is high for Bicoid and Caudal and low for Giant, Hunchback and Kruppel. It is worthwhile to note that considering only the information content, a naïve assumption would be that Hunchback and Kruppel have the highest specificity, but, when including the λ scaling factor, these two TFs display the lowest specificity.

In this context, one might ask if TF with low λ cannot distinguish well between different DNA words, where does the high information content of their motif come from? One hypothesis is that the methods used to determine TF specificity can potentially display technical biases. In fact, two different *in vitro* methods, SELEX (16) and bacterial one hybrid (39), lead to different PWM motifs for three of the TFs (Giant, Hunchback and Kruppel). When the motifs display higher information content (Giant in JASPAR and Hunchback and Kruppel in BDTNP), our method estimates a higher λ, which leads to lower specificities of the TFs. When the TFs display lower information content (Giant in BDTNP and Hunchback and Kruppel in JASPAR), our method estimates lower values for λ, which is consistent with the intuition that low information contents of the motifs will lead to low specificities. For the TFs that display similar PWM motifs in both sources (Bicoid and Caudal), we always estimate similar values for λ, which indicates that the specificity of the two TFs is given by the information content of the motifs.

Nevertheless, Supplementary Figures S9 and S10 show that the ChIP-seq profiles of Hunchback and Kruppel display some sharp peaks, which suggest that these two TFs display higher specificity than predicted by our approach. This contradicts our findings and one explanation for the few narrow ChIP-seq peaks is that these two TFs bind cooperatively to the genome. In this scenario, in the few narrow peaks for Hunchback and Kruppel, these TFs co-localize with co-factor(s) and previous studies identified that this is the case for both TFs; e.g. (17). This means that, by using our model, one could potentially underestimate the number of peaks in the binding profile.

Finally, we obtained the highest correlation and lowest mean squared error between the ChIP-seq profile and our estimate for the TFs that display the highest specificity (Bicoid and Caudal). Thus, our model performs best in the case of TFs that can discriminate better between strong and weak binding sites. Note that we observed the same result also when investigating the binding profiles genome-wide; see Figure 5. This reduction in the accuracy of our model for regions with weak binding is not a direct consequence of our model being an analytical approximation of the full statistical thermodynamics model, because even exact solutions to the full model display reduced accuracy for regions where TFs do not bind strongly; e.g. (16). It is worthwhile to note that regions with weaker binding seem to also have a lower chance of driving expression (61,68) and might potentially be experimental artefacts; e.g. (69).

### Additional factors that affect TF binding profiles

Our analytical model can recapitulate observed genome-wide binding profiles (e.g. for four of the TFs, the correlation is higher than 0.65) especially at the loci with strong binding, but there are several loci, where our model under/overestimates the ChIP-seq profile; see Figure 4 and Supplementary Figures S6–S10. In this contribution, we systematically investigated potential causes for these differences.

First, there is an inconsistency in the experimental data in the sense there are peaks in the ChIP-seq profile that are located in DNA inaccessible areas, e.g. there are peaks in the Bicoid ChIP-seq profile at *run*, *slp*, *eve*, *tll*, *gt*, *oc* loci that overlap with DNA that is marked as inaccessible; see Supplementary Figure S6. This indicates that either or both the DNA accessibility or the ChIP-seq data display some technical biases, e.g. (69,70), and, in these cases, the analytical model assumes that the DNA accessibility data are accurate and predict that there is no binding in DNA inaccessible areas. One solution is to use continuous data for DNA accessibility, where different areas display different levels of accessibility. When using continuous values for DNA accessibility data, we did not observe any improvements of our model's predictions. Nevertheless, we still observed ChIP-seq peaks for all five TFs that were overlapping with regions with reduced or no accessibility, thus, indicating the one or both data sets (ChIP-seq or DNase I) contain experimental biases; e.g. (69–71).

Alternatively, the underestimation of the peak height may be caused by PWM choice. Using motifs from the JASPAR database (39), we observed lower values for the correlation and higher values for the mean squared error compared to the case of using the PWMs from BDTNP (16). In addition, when we used different PWMs (from the JASPAR database) we found different estimates for the number of molecules that best explain the ChIP-seq data, but these values were within the same range (2000–10 000 molecules). This suggests that our estimates for the amount of bound TFs are not the exact values, but rather an estimate of the order of magnitude for the number of molecules that are bound specifically to the DNA.

In this manuscript, we aim to deconvolute the contributions of different factors to the binding profiles of TFs. One of the most important factors that contribute to the binding profiles is the binding energy between the TF and the DNA words. Previous work (21,72,73) showed that the binding energy between a TF and the DNA is proportional to the PWM score and, thus, the binding energy can be approximated by a scaled PWM score (*E*_*i*_ = *w*_*i*_/λ). In order to avoid introducing the effects of ‘other factors’ in the binding energy estimation, one should consider that the PWM is representing only the binding frequency between the TF and the DNA words independent of other factors. Inferring the PWM motif from the ChIP-seq peaks would assume that DNA accessibility, TF cooperativity, crowding of molecules on the DNA, histone marks and others will affect the PWM. Whilst by using a PWM derived from ChIP-seq data we might lead to better predictions of the binding profiles, we would not be able to distinguish between the real sources that drive the genomic occupancy and their relative contribution. This is the rationale for testing PWMs derived from BDTNP (16) and JASPAR(39) and not investigating the case of the PWMs derived from ChIP-seq data.

Furthermore, we do not consider every aspect related to the binding of TFs to the genome, but binding energy (PWM scores and λ), TF abundance and DNA accessibility are sufficient to explain most of the characteristics of the binding profiles for the TFs analysed in this study (Bicoid, Caudal, Giant, Hunchback and Kruppel). One aspect that our model does not include is cooperative binding to the DNA. Previous studies have shown that TF cooperativity can significantly impact the binding of TFs (13,18–20,74,75) and that cooperativity can explain TF genomic binding (17). However, it was found that the five TFs considered in this study display negligible or no cooperative interactions between them (16,17), but these TFs seem to display cooperative interactions with other TFs (17,76,77). For example, the Bicoid binding profile seems to be significantly influenced by the maternally contributed factor Zelda, where the presence of Zelda increases the binding of Bicoid at the majority of loci and decreases it at a small set of loci (76). Modelling these binding profiles assuming cooperativity with other TFs could potentially improve our model predictions, but this requires further systematic investigation and will be left to future research.

Our model does not implement competitive binding directly. Other models, e.g. (16), allow one to model the competition between TFs explicitly, but the fact that we obtained similar correlation between the predicted profile and the ChIP-seq data indicates that there is negligible binding competition between the five TFs analysed in this study as also shown in (67).

Finally, we would like to point out that whilst our model was applied to a ChIP-seq data set, it could also be used to investigate ChIP-chip and ChIP-exo (53) data sets as long as the appropriate length distribution of DNA fragments is included in the model (see the Materials and Methods section).

## AVAILABILITY

The R scripts used to perform the analysis can be downloaded from https://github.com/nrzabet/ChIPseqProfile.

## SUPPLEMENTARY DATA

Supplementary Data are available at NAR Online.

SUPPLEMENTARY DATA
