# Supplementary material for: Estimating binding properties of transcription factors from genome-wide binding profiles
Source: Nucleic Acids Res. 2014 Nov 28;43(1):84–94. doi: 10.1093/nar/gku1269 (PMC4288167; doi:10.1093/nar/gku1269)
Supplement: SUPPLEMENTARY DATA [file supp_43_1_84__index.html]

Estimating binding properties of transcription factors from genome-wide binding profiles — SUPPLEMENTARY DATA 

# Estimating binding properties of transcription factors from genome-wide binding profiles

## SUPPLEMENTARY DATA

**Files in this Data Supplement:**

- SUPPLEMENTARY DATA
