# Supplementary material for: Estimating binding properties of transcription factors from genome-wide binding profiles
Source: Nucleic Acids Res. 2014 Nov 28;43(1):84–94. doi: 10.1093/nar/gku1269 (PMC4288167; doi:10.1093/nar/gku1269)
Supplement: SUPPLEMENTARY DATA [file supp_gku1269_nar-02180-n-2014-File002.pdf]

Supplementary Material to:

Estimating binding properties of transcription factors from genome-wide binding profiles

Nicolae Radu Zabet[1,2,*] and Boris Adryan[1,2,†]

[1]Cambridge Systems Biology Centre, University of Cambridge, Tennis Court Road, Cambridge CB2 1QR, UK

[2]Department of Genetics, University of Cambridge, Downing Street, Cambridge CB2 3EH, UK

[*]Email: n.r.zabet@gen.cam.ac.uk [†]Email: ba255@cam.ac.uk

S1 Derivation of the analytical model

Here, we derive an analytical solution for the statistical thermodynamics model, which makes it feasible to investigate genome-wide binding profiles of TFs, rather than being restricted to much shorter loci.

S1.1 Binding energy between the TF and the DNA

The amount of time a TF is bound to a specific site can be computed using the binding energy as [1]:

$$\tau_j = \tau_0 \exp\left(-E_j\right) = \tau_0 \exp\left(\frac{1}{\lambda} w_j\right) \tag{S1}$$

where j is the genomic coordinate of the site, τ_0 is a scaling factor associated with each TF species (which takes into account an absolute strength between the TF and the DNA) and E_j is the binding energy of site j. Note that the binding energy can be approximated by the PWM score $E_j = \frac{1}{\lambda} w_j$, where $1/\lambda$ is a scaling factor which quantifies the penalty for differences from the preferred site [2, 3] and w_j the PWM score at position j.

We want to investigate the accuracy of our analytical model and, thus, we compare its results with the results from a detailed statistical thermodynamics model, as computed with GRiP [4–7]; see section S2. As previously shown in [8], for highly abundant TFs, the estimation of the occupancy based on the PWM alone is not sufficient to accurately predict the profile found in simulations (and also by a simple statistical thermodynamics framework); see Figure S2. Note that in Figure S2 we do not simulate the 1D random walk on the DNA, while in Figure 3 in [8] the 1D random walk on the DNA is considered.

S1.2 The number of bound molecules to the DNA

We used our analytical solution for the statistical thermodynamics framework to include TF abundance in the model. Given that a TF has N molecules bound to the DNA, the statistical weight that a site is unoccupied by a molecule can be written as [9–12]

$$Z\left(N\right) = \overbrace{\frac{\left(L \cdot n\right)!}{N!\left(L \cdot n - N\right)!}}^{\text{number of arrangements}} \times \underbrace{\exp\left(-E^{ns}\right)}_{\text{Boltzmann weight}} \tag{S2}$$

where E_x^{ns} is the binding free energy when the TF is non-specifically bound to the DNA (i.e. not to the target site), L represents the total number of available sites (which can be approximated by the length of the DNA segment) and n is the ploidy level (the number of copies of the genome, e.g. for diploid genomes $n = 2$). The total statistical weight is given by the sum of the statistical weight when the site is unoccupied and the statistical weight when the site is occupied.

$$Z_{total}(N) = \overbrace{Z(N)}^{\text{the site is empty}} + \overbrace{Z(N-1)\exp(-E^s)}^{\text{the site is occupied}} \tag{S3}$$

where E^s binding free energy at the target site (where the TF is bound specific). Altogether the probability that the target site is occupied is given by the ratio between the statistical weight of the site being occupied over the total statistical weight.

$$P^{bound} = \frac{Z(N-1)\exp(-E^s)}{Z_{total}(N)} = \frac{1}{1 + \frac{Z(N)}{Z(N-1)\exp(-E^s)}} \tag{S4}$$

This can be reduced to [10] :

$$P^{bound}(E_j, N) = \frac{1}{1 + \frac{L\cdot n - N + 1}{N}\exp[(E^s - E^{ns})]} = \frac{1}{1 + \frac{(L\cdot n - N+1)\exp(-E^{ns})}{N}\exp(E^s)} \tag{S5}$$

Given the size of the genome and the range of TF abundances reported in the literature, we can assume that the number of available sites is much larger than the number of bound molecules ($L \cdot n \gg N$) and, thus, $(L \cdot n - N + 1) \approx L \cdot n$ [10]. In our model, we do not want to be constrained to only two binding energy levels (the binding energy for non-specific sites and for specific sites), but rather consider the full binding energy spectrum. If E^{ns} represents the binding energy at other sites (not the current one), then $L \cdot n \cdot \exp(-E^{ns})$ represents the average waiting time on the genome and can be approximated by $L \cdot n \cdot \langle \exp(-E_i) \rangle_i = L \cdot n \cdot \left\langle \exp\left(\frac{1}{\lambda} w_i\right) \right\rangle_i$ [13]. This leads to the following probability of site j being occupied by a TF molecule:

$$P_j^{bound}(\lambda, w, N) = \frac{1}{1 + \frac{1}{N} L \cdot n \cdot \left\langle \exp\left(\frac{1}{\lambda} w_i\right)\right\rangle_i \exp\left(-\frac{1}{\lambda} w_j\right)} = \frac{N \exp\left(\frac{1}{\lambda} w_j\right)}{N \exp\left(\frac{1}{\lambda} w_j\right) + L \cdot n \cdot \left\langle \exp\left(\frac{1}{\lambda} w_i\right)\right\rangle_i} \tag{S6}$$

Figure S3 shows that, by including the TF abundance in our model, the estimate of the occupancy obtained in the simulations improves significantly; compare Figure S2 to Figure S3.

One approximation of our model is that we assume the mean value instead of the full distribution of PWM scores [14]. However, we found that, by using this approximation (the mean value of the PWM scores) [13], the difference between the analytical and numerical results are negligible; see Figure S3. This approximation also holds in the case of TFs with lower specificity as we show in the *Results* section of the main manuscript.

S1.3 Including DNA accessibility data

To apply our model to eukaryotic systems we need to include DNA accessibility as a parameter. The probability that a site j is bound, in the case of N bound molecules to the DNA and in presence of DNA accessibility data, can be rewritten as

$$P_j^{bound}(\lambda, w, N, a) = \frac{N \cdot a_j \cdot \exp\left(\frac{1}{\lambda} w_j\right)}{N \cdot a_j \cdot \exp\left(\frac{1}{\lambda} w_j\right) + L \cdot n \cdot \left\langle a_i \exp\left(\frac{1}{\lambda} w_i\right)\right\rangle_i} \tag{S7}$$

where a_j the probability that site j on the genome is in accessible chromatin.

S1.4 Analysing ChIP-seq data with the analytical model

To test the accuracy of our analytical model, we compare its estimated profile to ChIP-seq data and, thus, we need to convert the prediction of the analytical model to a profile of genomic occupancy. Given, C_j as the experimentally determined ChIP-seq signal at position j on the genome, the equivalent occupancy based on the analytical estimate is [13]

$$A(j, \lambda, w, N) = B + (M - B) \times P_j^{bound} \tag{S8}$$

where $B = \langle C \rangle$ is the background and $M = \max(C)$ is the maximum of the ChIP-seq signal.

S1.5 Modelling DNA accessibility data

Following the approach in [15], we modelled the probability of a site being accessible as

$$a_j = \frac{1}{1 + \exp(-\beta \cdot DD_j + \alpha)} \tag{S9}$$

where DD_j is the DNase I read density and α and β are scaling parameters, which were estimated to be $\alpha = 6.008$ and $\beta = 0.207$ in [15].

S2 Computing the equilibrium binding profile with GRiP

To obtain the profile generated with a statistical thermodynamics approach, we performed stochastic simulations with our previously published computational tool GRiP [4–6] that simulates the facilitated diffusion of TFs [16]. In GRiP, all the molecules in the system are represented explicitly, along with steric hindrance effects. Three-dimensional diffusion is simulated using the Master Equation, an approximation which was previously proven to provide accurate results [17]. The simulations were restricted to binding and unbinding to/from the DNA (we removed the one-dimensional random walk on the DNA from the simulations), which means that the results are equivalent to the ones predicted by the classical statistical thermodynamics framework. To compare the profiles generated by simulations (S) and the ones generated by the analytical approximation (A), we normalise all profiles to the highest values.

When comparing the analytical model to the results computed by GRiP, we considered the case of lac repressor (lacI), a well studied bacterial TF and 20 Kbp of *E.coli* DNA (355000..375000 locus in the *E.coli* K-12 genome). For lacI, we considered a motif constructed based on the three high-affinity sites of lacI in [6]; see Figure S1. The default parameters for the simulations with GRiP are listed in Table S1. Furthermore, we considered four cases with respect to lacI abundance and the set of parameters selected for each case ensured that each site is visited on average approximately 2000 times; see Table S2. We performed 50 independent simulations for each set of parameters and computed the occupancy as time average in each simulation. Overall, this resulted in each site being visited on average 10^5 times.

Finally, to generate the ChIP profiles we used the method described by [15], where a mean segment length of 150 bp, a standard deviation of 150 bp and no smoothing was used; the R implementation of this method is described in [8]. The difference between the simulations and our analytical formula was quantified by: (i) the Pearson coefficient of correlation and (ii) the mean squared error.

**parameter**	**lacI**	**notation**
copy number	see Table S2	n_x
motif sequence	see Figure S1	
energetic penalty for mismatch	$1K_BT$	ε_x^*
nucleotides covered on left	0 bp	δ_x^{left}
nucleotides covered on right	0 bp	δ_x^{right}
association rate to the DNA	see Table S2	k_x^a
unbinding probability	1.0	P_x^{unbind}
probability to slide left	0	P_x^{left}
probability to slide right	0	P_x^{right}
probability to dissociate completely when unbinding	1.0	P_x^{jump}
time bound at the target site	$1.18E-6$ s	τ_x^0
the size of a step to left	1 bp	
the size of a step to right	1 bp	
variance of repositioning distance after a hop	1 bp	σ^2_{hop}
the distance over which a hop becomes a jump	100 bp	d_{jump}

Table S1: *TF species default parameters*

**lacI copy number**	**association rate to the DNA**	**simulation time**	**bound molecules**
100	2700	150 s	92
1000	3100	15 s	930
10000	4000	1.2 s	8852
100000	20000	0.075 s	91784

Table S2: *The parameters for the six cases of lacI abundance.* The association rate was selected so that on average 90% of the time the TF molecules are bound to the DNA.

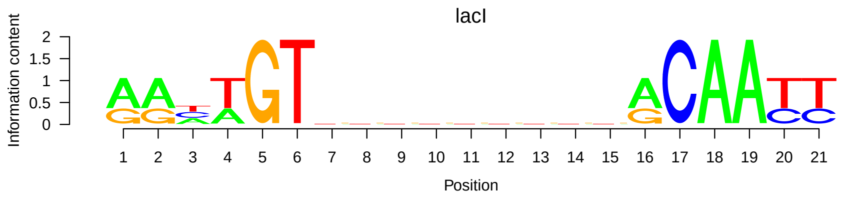

Figure S1: *Sequence logo for lacI.* [6]

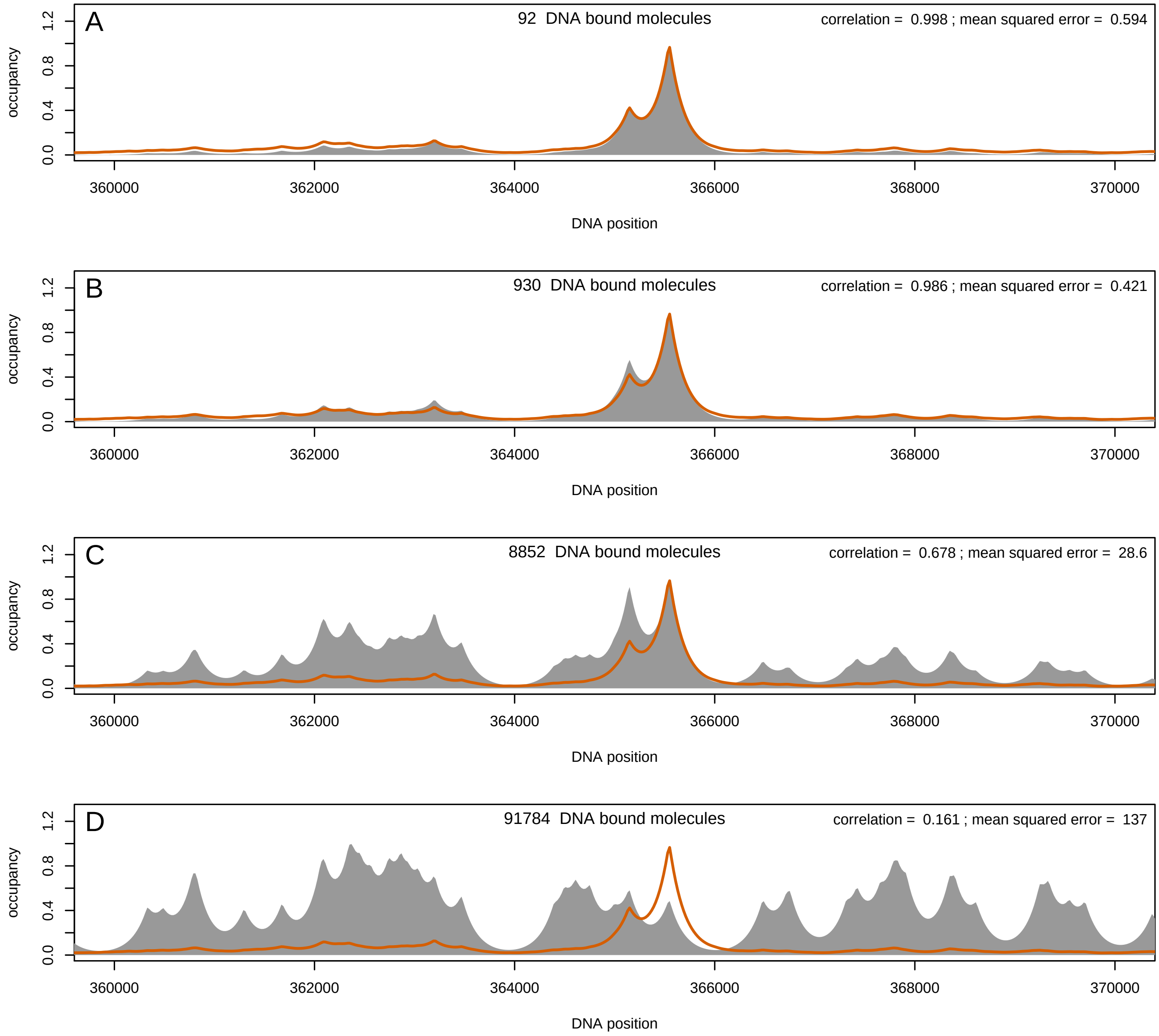

Figure S2: *Estimating the occupancy in the simulations (or statistical thermodynamic model) based on the PWM score alone.* We considered the case of the lac repressor and 20 Kbp of DNA, which contain the O_1 site. In each chart, the filled region is the occupancy predicted base on the simulations (S), and the solid line is the occupancy estimated by the analytical solution; see equation S1. We considered various lacI abundances: (A) 100 lacI molecules (leading to 92 lacI molecules being bound to the DNA), (B) 1000 lacI molecules (i.e. 930 lacI molecules), (C) 10000 lacI molecules (i.e. 8852 lacI molecules) and (D) 100000 lacI molecules (i.e. 91784 lacI molecules). For each set of parameters, we consider $X = 50$ independent simulations. We considered only the sites that have the binding energy of at least 70% of the highest value (the strongest 81 sites). We converted the single nucleotide resolution into expected occupancy profiles as proposed in [15], with a mean of 150 bp and a standard deviation of 150 bp; see also [8]. The inset in each panel indicates the Pearson coefficient of correlation and the mean squared error between the occupancy computed by the analytical model and the occupancy computed from the simulations, which is equivalent to the occupancy generated by the full statistical thermodynamics model. The x-axis represents the genomic location.

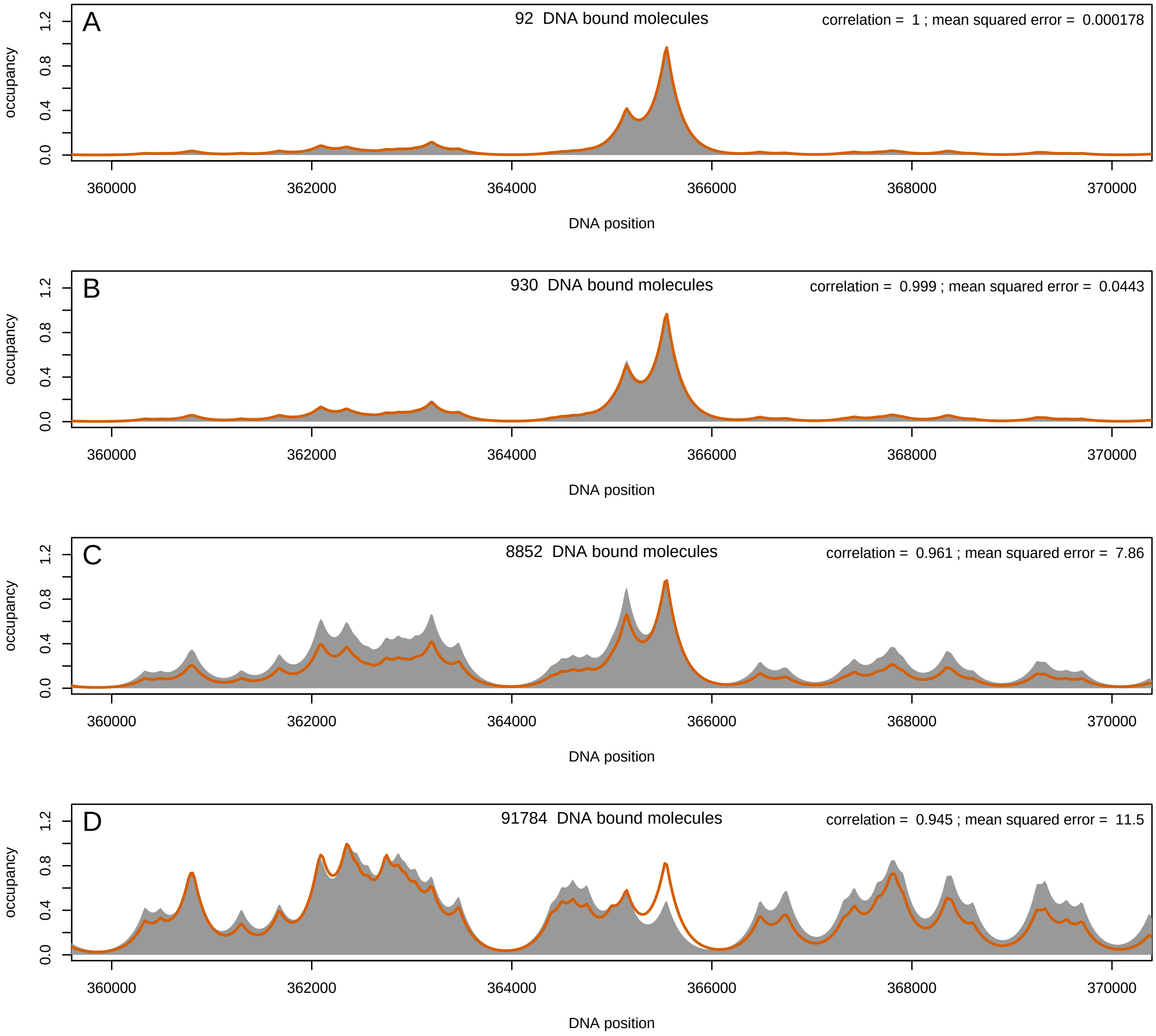

Figure S3: *Estimating the occupancy in the simulations (or statistical thermodynamic model) based on the PWM and TF abundance.* This is the same as Figure S2, except that equation (S6) was used to compute the occupancy, solid line.

S3 Data sets

Symbol	Gene name	Locus coordinates	Length
croc	crocodile	chr3L:21,461,001-21,477,000	16 Kb
cnc	cap-n-collar	chr3R:19,011,001-19,024,000	13 Kb
slp	sloppy paired	chr2L:3,820,001-3,840,000	20 Kb
kni	knirps	chr3L:20,683,260-20,695,259	12 Kb
hkb	huckebein	chr3R:169,001-181,000	12 Kb
D	Dichaete	chr3L:14,165,001-14,179,000	24 Kb
prd	paired	chr2L:120,77,501-12,095,500	18 Kb
H	hairy	chr3L:8,656,154-8,682,153	26 Kb
eve	even skipped	chr2R:5,860,693-5,876,692	16 Kb
cad	caudal	chr2L:20,767,501-20,786,500	19 Kb
oc	ocelliless	chrX:8,518,001-8,550,000	32 Kb
opa	odd paired	chr3R:670,001-696,000	26 Kb
ftz	fushi tarazu	chr3R:2,682,501-2,696,500	14 Kb
gt	giant	chrX:2,317,878-2,330,877	13 Kb
hb	hunchback	chr3R:4,513,501-4,531,500	18 Kb
Kr	Kruppel	chr2R:21,103,924-21,118,923	15 Kb
odd	odd skipped	chr2L:3,603,001-3,613,000	10 Kb
run	runt	chrX:20,548,001-20,570,000	22 Kb
fkh	forkhead	chr3R:24,396,001-24,420,000	24 Kb
tll	tailless	chr3R:26,672,001-26,684,000	12 Kb
os	outstretched	chrX:18,193,001-18,208,000	15 Kb

Table S3: *Genes and coordinates for analysed sets loci.* These are the same *loci* as considered in [15].

S4 Analysis of the ChIP-seq profiles

S4.1 Heatmaps for GT, HB and KR

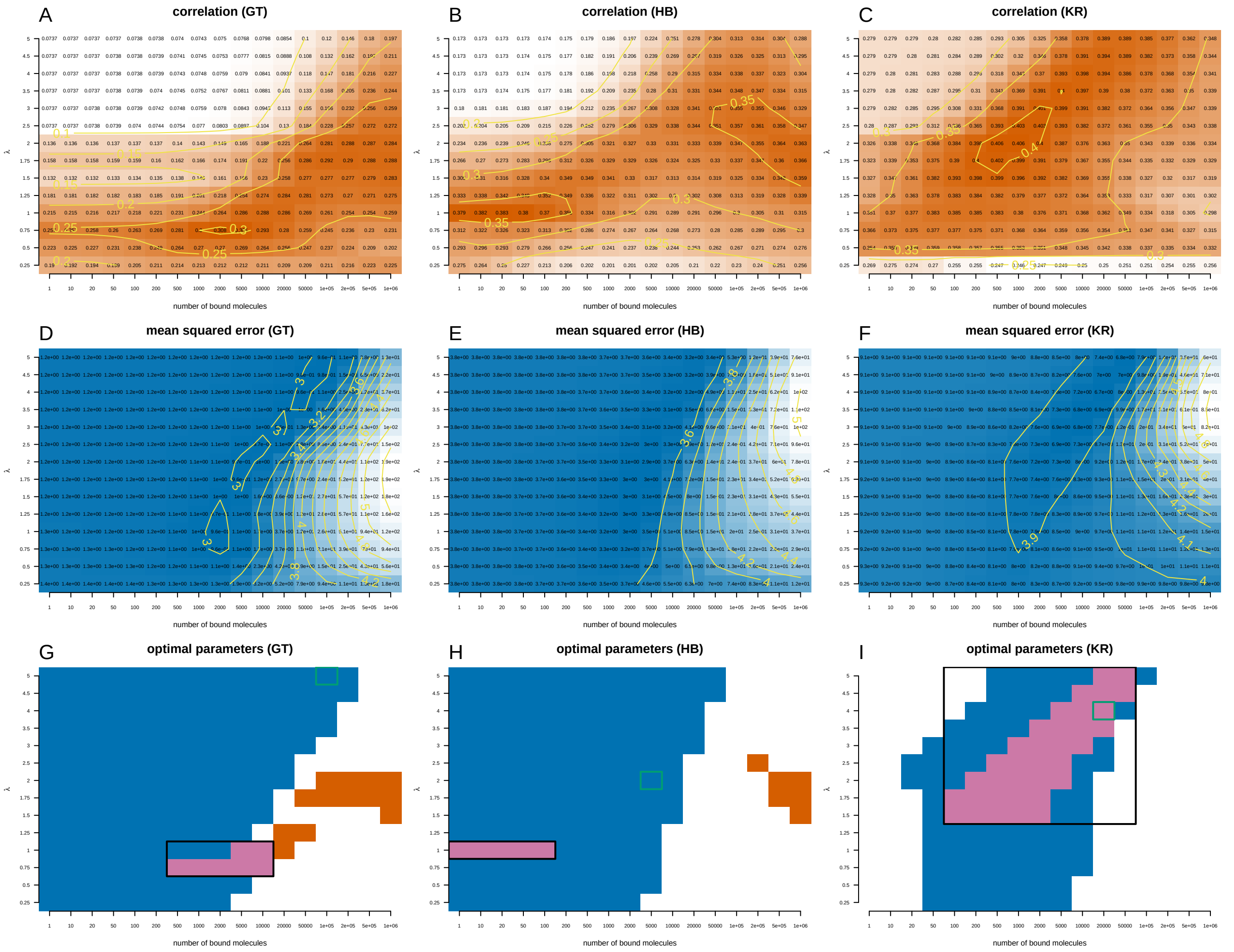

Figure S4: *Quantifying the distances between Giant, Hunchback and Kruppel ChIP-seq profiles and the profiles derived with the analytical model.* We plotted heatmaps for the correlation $(A - C)$ and mean squared error $(D - F)$ between the analytical model and the ChIP-seq profile of Giant (A, D), Hunchback (B, E) and Kruppel (C, F). We computed these values for different sets of parameters: $N \in [1, 10^6]$ and $\lambda \in [0.25, 5]$. We considered only the sites that have a PWM score higher than 70% of the difference between the lowest and the highest score. $(A - C)$ Orange colour indicates high correlation between the analytical model and the ChIP-seq profile, while white colour low correlation. $(D - F)$ Blue colour indicates low mean squared error between the analytical model and the ChIP-seq profile, while white colour high mean squared error. $(G - I)$ We plotted the regions where the mean squared error is in the lower 12% of the range of values (blue) and the correlation is the higher 12% of the range of values (orange). With green rectangle we marked the optimal set of parameters in terms of mean squared error and with a black rectangle the intersection of the parameters for which the two regions intersect.

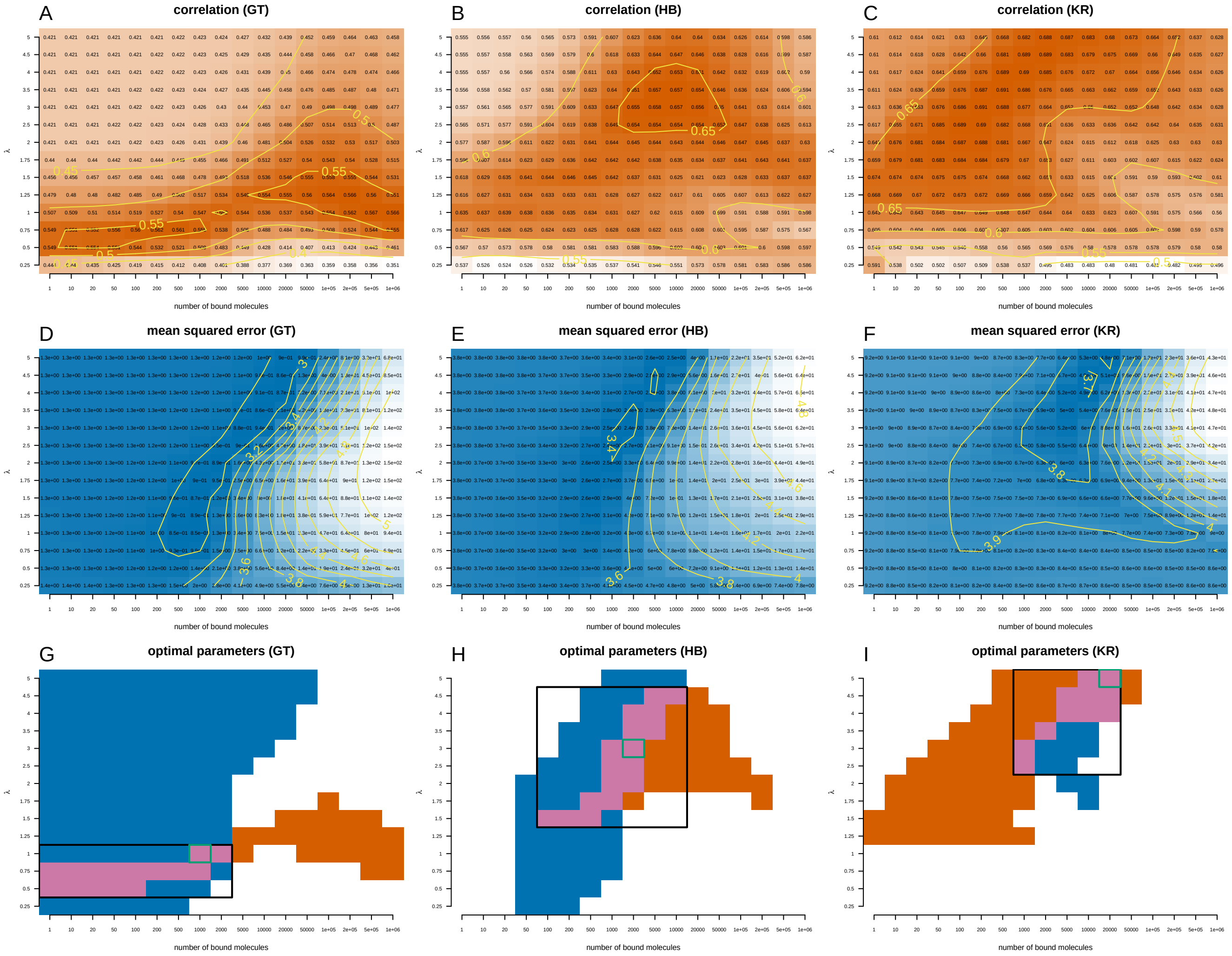

Figure S5: *Quantifying the distances between Giant, Hunchback and Kruppel ChIP-seq profiles and the profiles derived with the analytical model which includes DNA accessibility data.* This is the same as Figure S4, except that we included binary DNA accessibility data in the analytical model.

S4.2 Profiles for all 21 loci

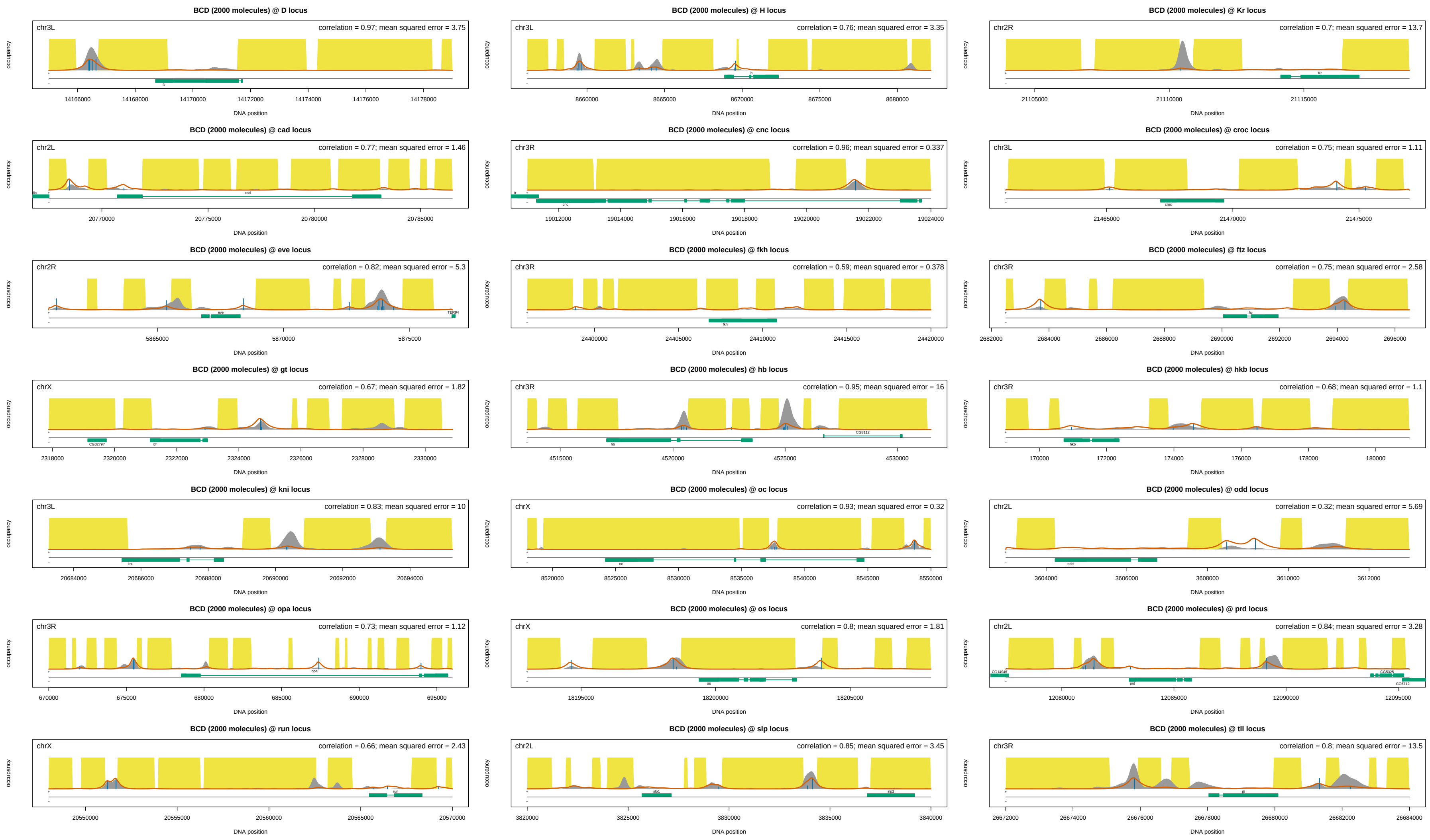

Figure S6: *Binding profiles for Bicoid at all 21 loci.* The grey shading represents a ChIP-seq profile, the red line represents the prediction of the analytical model, the yellow shading represents the inaccessible DNA and the vertical blue lines represent the percentage of occupancy of the site (we only displayed sites with an occupancy higher than 5%). We considered the optimal set of parameters for Bicoid (2000 *molecules* and $\lambda = 1.25$).

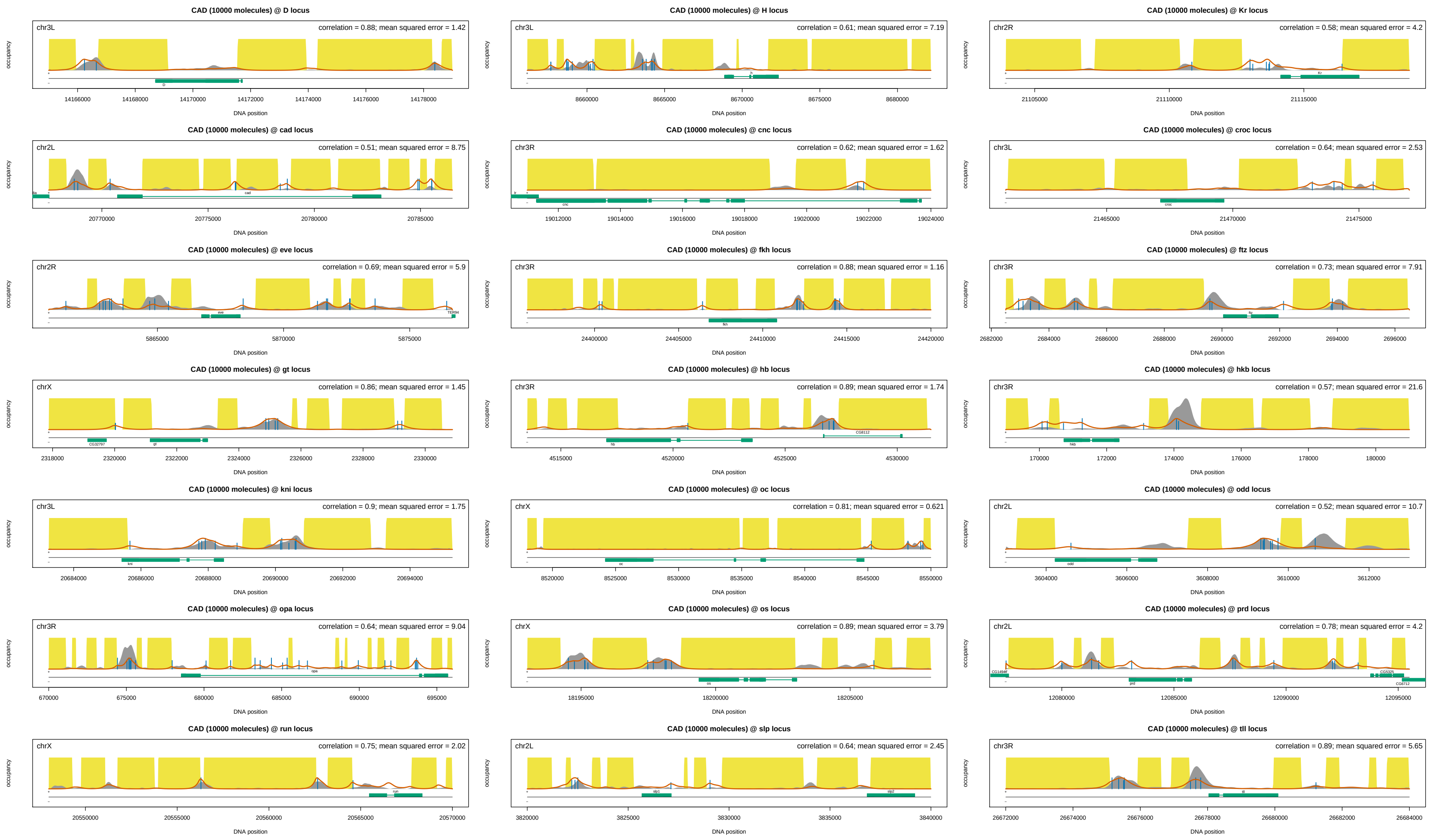

Figure S7: *Binding profiles for Caudal at all 21 loci.* The grey shading represents a ChIP-seq profile, the red line represents the prediction of the analytical model, the yellow shading represents the inaccessible DNA and the vertical blue lines represent the percentage of occupancy of the site (we only displayed sites with an occupancy higher than 5%). We considered the optimal set of parameters for Caudal (10000 *molecules* and $\lambda = 1.25$).

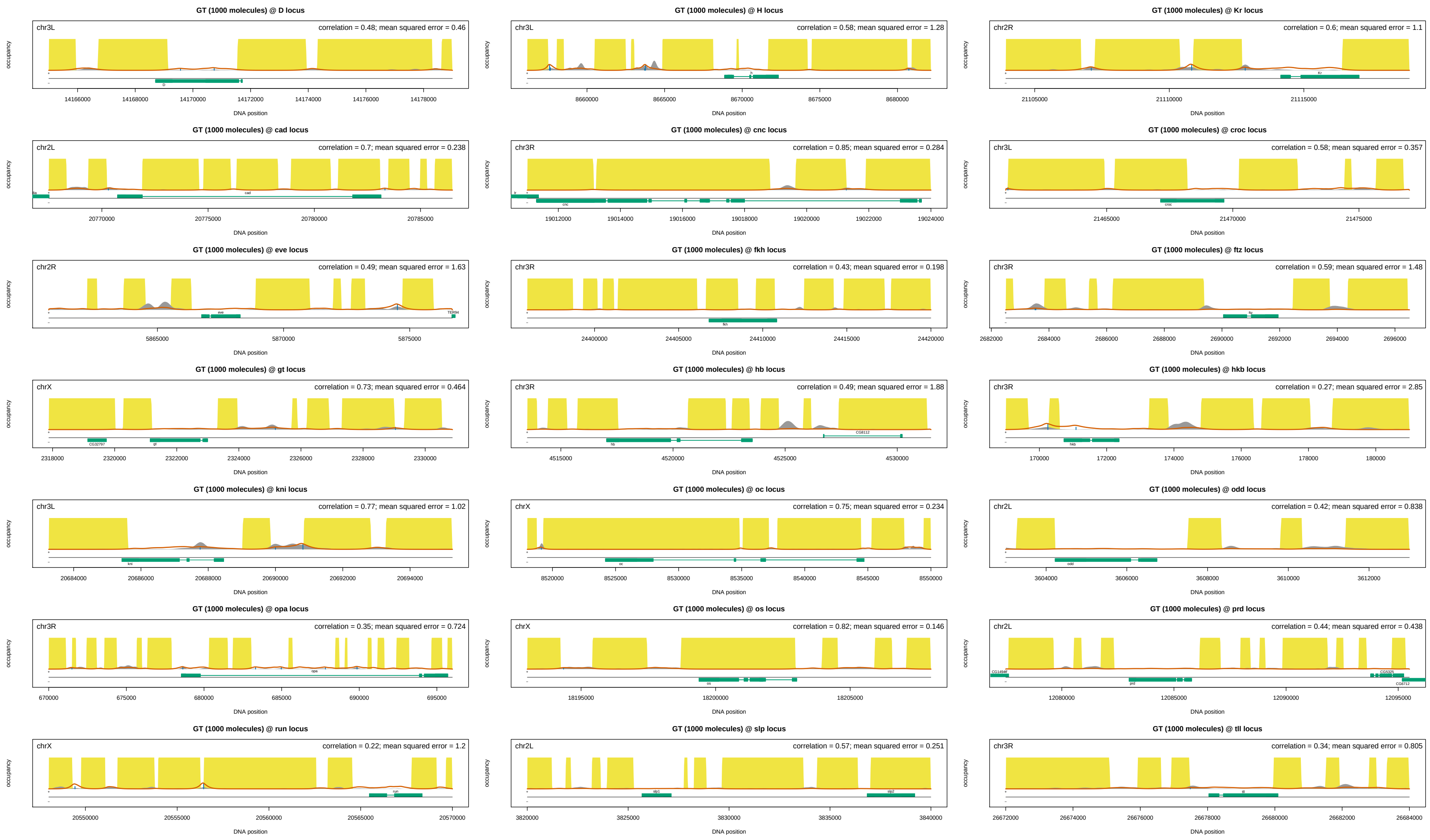

Figure S8: *Binding profiles for Giant at all 21 loci.* The grey shading represents a ChIP-seq profile, the red line represents the prediction of the analytical model, the yellow shading represents the inaccessible DNA and the vertical blue lines represent the percentage of occupancy of the site (we only displayed sites with an occupancy higher than 5%). We considered the optimal set of parameters for Giant (1000 *molecules* and $\lambda = 1.00$).

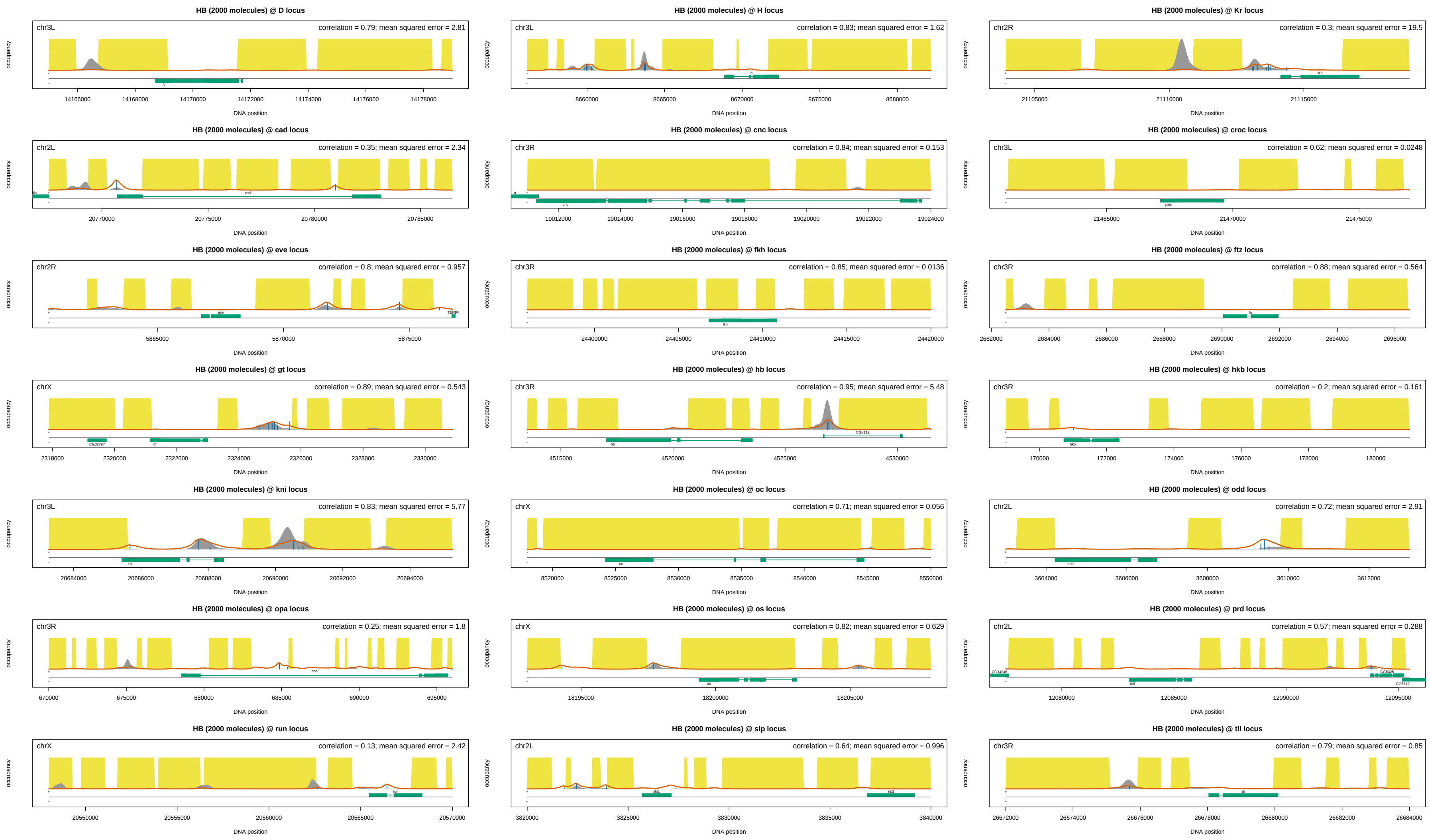

Figure S9: *Binding profiles for Hunchback at all 21 loci.* The grey shading represents a ChIP-seq profile, the red line represents the prediction of the analytical model, the yellow shading represents the inaccessible DNA and the vertical blue lines represent the percentage of occupancy of the site (we only displayed sites with an occupancy higher than 5%). We considered the optimal set of parameters for Hunchback (2000 *molecules* and $\lambda = 3.00$).

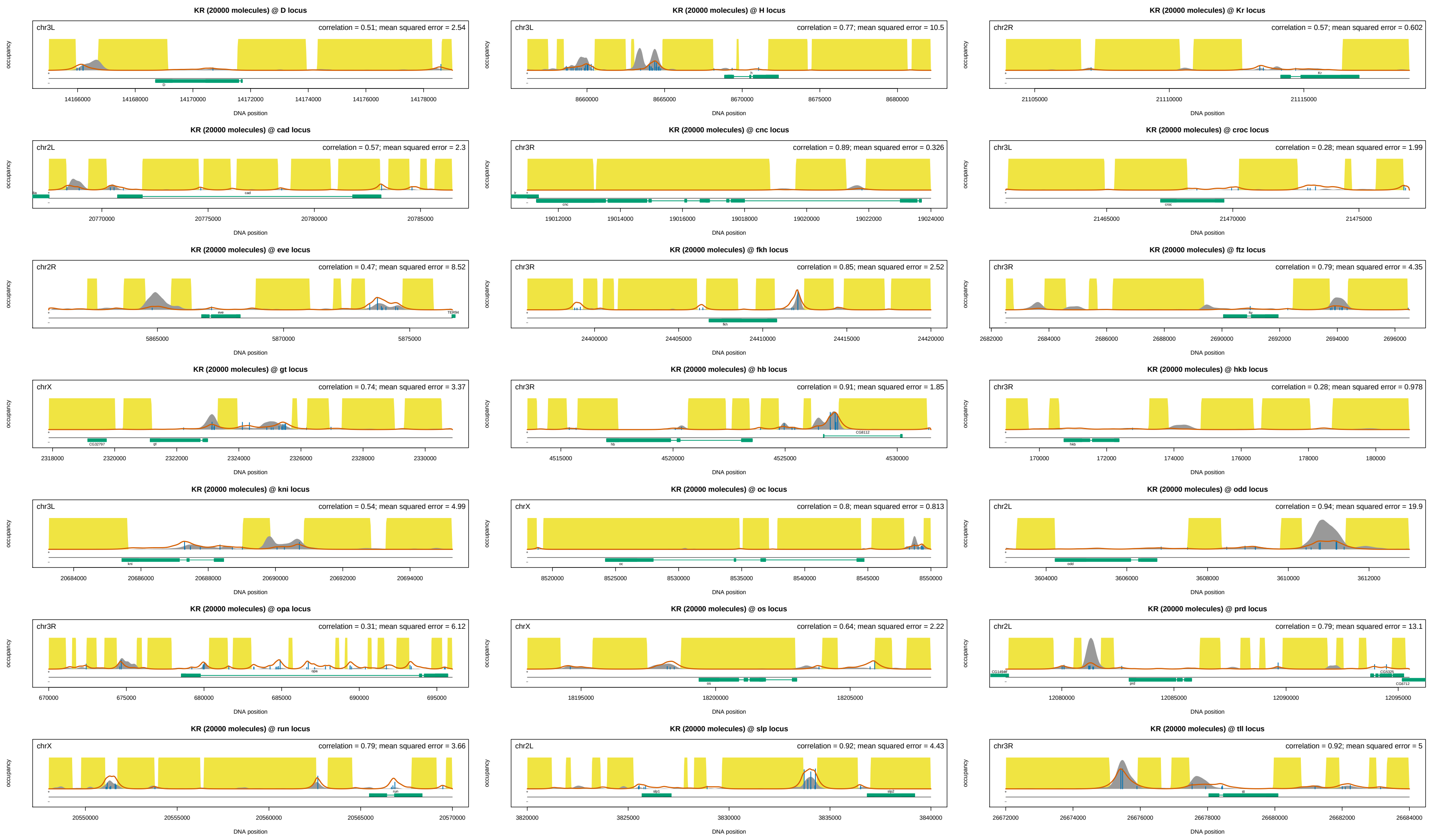

Figure S10: *Binding profiles for Kruppel at all 21 loci.* The grey shading represents a ChIP-seq profile, the red line represents the prediction of the analytical model, the yellow shading represents the inaccessible DNA and the vertical blue lines represent the percentage of occupancy of the site (we only displayed sites with an occupancy higher than 5%). We considered the optimal set of parameters for Kruppel (20000 *molecules* and $\lambda = 5.00$).

S4.3 Including weak binding into the model

	N	λ	MSE	ρ
BCD	2000	1.25	4.40 (4.40)	0.77 (0.77)
CAD	10000	1.25	5.03 (5.03)	0.73 (0.75)
GT	1000	1.00	0.85 (0.85)	0.55 (0.57)
HB	1000	2.50	2.40 (2.40)	0.64 (0.66)
KR	2000	3.00	5.46 (5.46)	0.66 (0.69)

Table S4: *Set of parameters that minimises the difference between the ChIP-seq profile when including weak binding.* The analytical model includes binary DNA accessibility data (the accessibility of any site can be either 0 or 1 depending on whether the site is accessible or not). We also listed the values for the mean squared error (MSE) and correlation (ρ). The values in the parentheses represent the minimum mean squared error and the maximum correlation. We considered only the sites that have a PWM score higher than 30% of the distance between the lowest and the highest score.

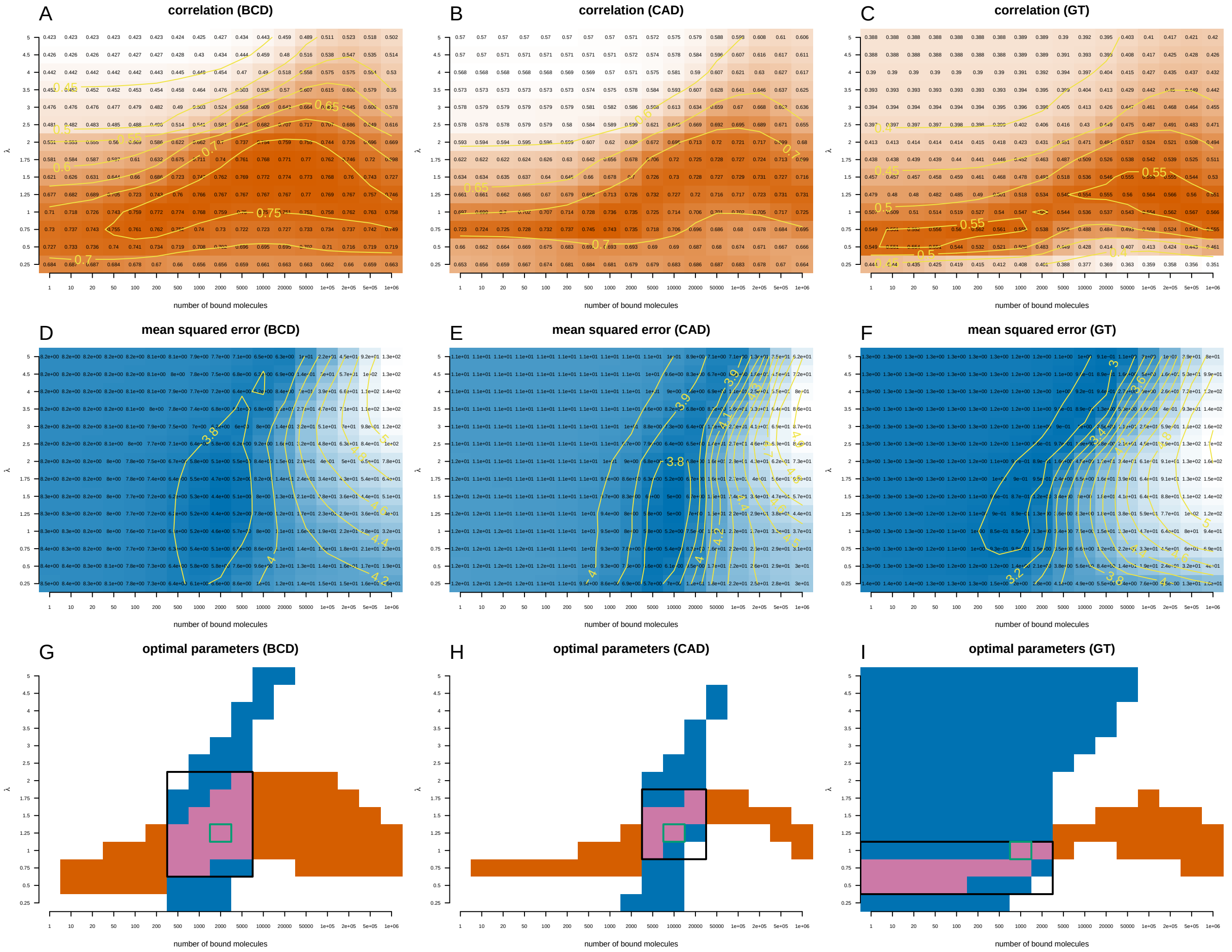

Figure S11: *The influence of weak binding on Bicoid, Caudal and Giant ChIP-seq profiles.* We plotted heatmaps for the correlation $(A-C)$ and mean squared error $(D-F)$ between the analytical model and the ChIP-seq profile of Bicoid (A, D), Caudal (B, E) and Giant (C, F). The analytical model includes binary DNA accessibility data (the accessibility of any site can be either 0 or 1 depending on whether the site is accessible or not). We computed these values for different sets of parameters $N \in [1, 10^6]$ and $\lambda \in [0.25, 5]$. Colour code as above. Here, only considering sites that have a PWM score higher than 30% of the difference between the lowest and the highest score. $(G-I)$ We plotted the regions where the mean squared error is in the lower 12% of the range of values (blue) and the correlation is the higher 12% of the range of values (orange). With green rectangle we marked the optimal set of parameters in terms of mean squared error and with a black rectangle the intersection of the parameters for which the two regions intersect.

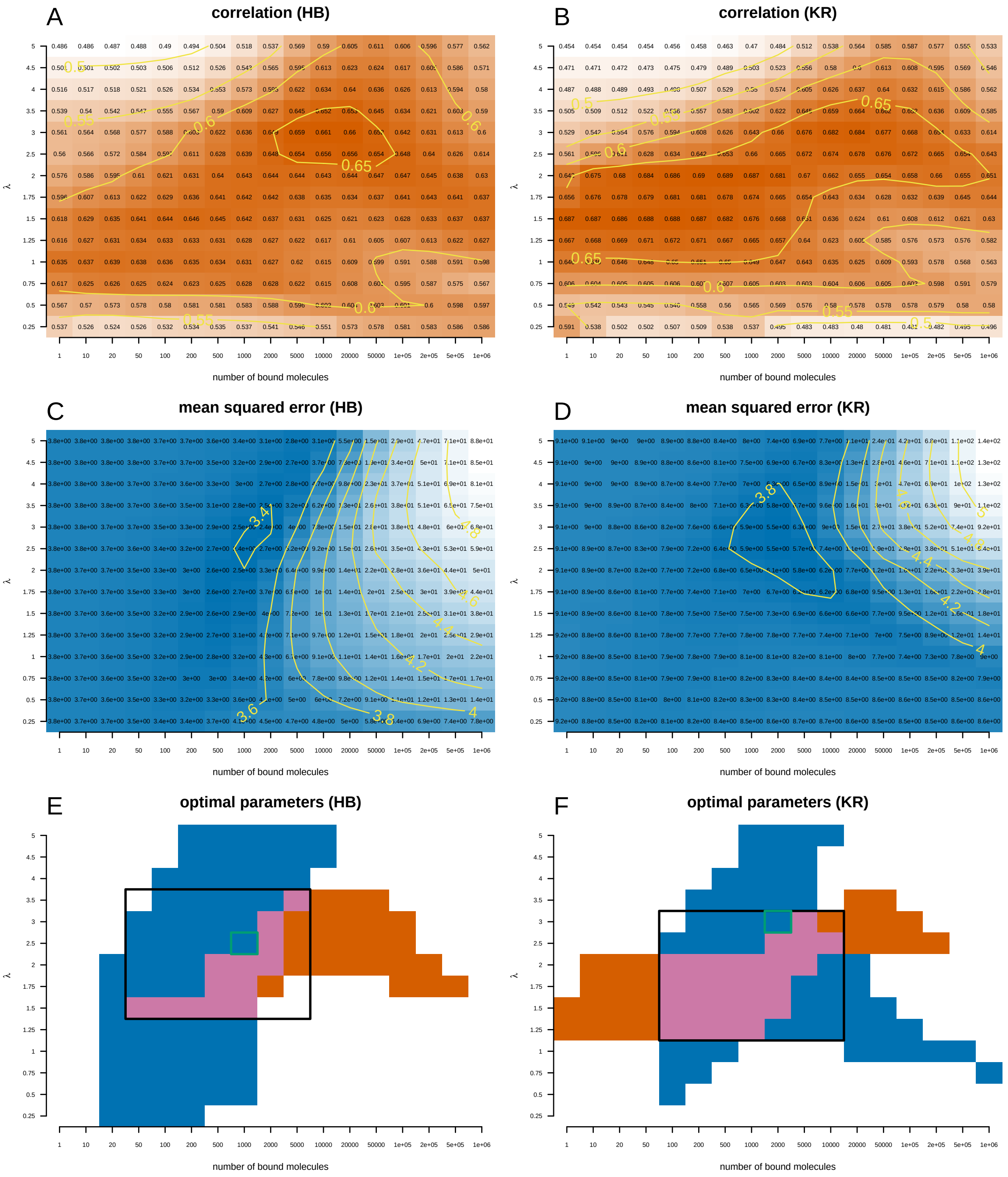

Figure S12: *The influence of weak binding on Hunchback and Kruppel ChIP-seq profiles.* We plotted heatmaps for the correlation (A) and (B) and mean squared error (C) and (D) between the analytical model and the ChIP-seq profile of Hunchback (A, C) and Kruppel (B, D). The analytical model includes binary DNA accessibility data (the accessibility of any site can be either 0 or 1 depending on whether the site is accessible or not). We computed these values for different sets of parameters: $N \in [1, 10^6]$ and $\lambda \in [0.25, 5]$. Colour code as above. PWM filtering as in Figure S11. (E, F) We plotted the regions where the mean squared error is in the lower 12% of the range of values (blue) and the correlation is the higher 12% of the range of values (orange). With green rectangle we marked the optimal set of parameters in terms of mean squared error and with a black rectangle the intersection of the parameters for which the two regions intersect.

S4.4 Continuous DNA accessibility data

	N	λ	MSE	ρ
BCD	500	1.25	4.47 (4.47)	0.77 (0.78)
CAD	2000	0.75	5.35 (5.35)	0.72 (0.75)
GT	200	1.00	0.83 (0.83)	0.54 (0.57)
HB	1000	3.50	2.38 (2.38)	0.65 (0.66)
KR	5000	5.00	4.81 (4.81)	0.68 (0.69)

Table S5: *Set of parameters that minimises the difference between the ChIP-seq profile in the case of continous accessibility data.* The analytical model includes continous DNA accessibility data by using equation (S9) to compute the probability that a site is accessible. We also listed the values for the mean squared error (MSE) and correlation (ρ). The values in the parentheses represent the minimum mean squared error and the maximum correlation. We considered only the sites that have a PWM score higher than 70% of the distance between the lowest and the highest score.

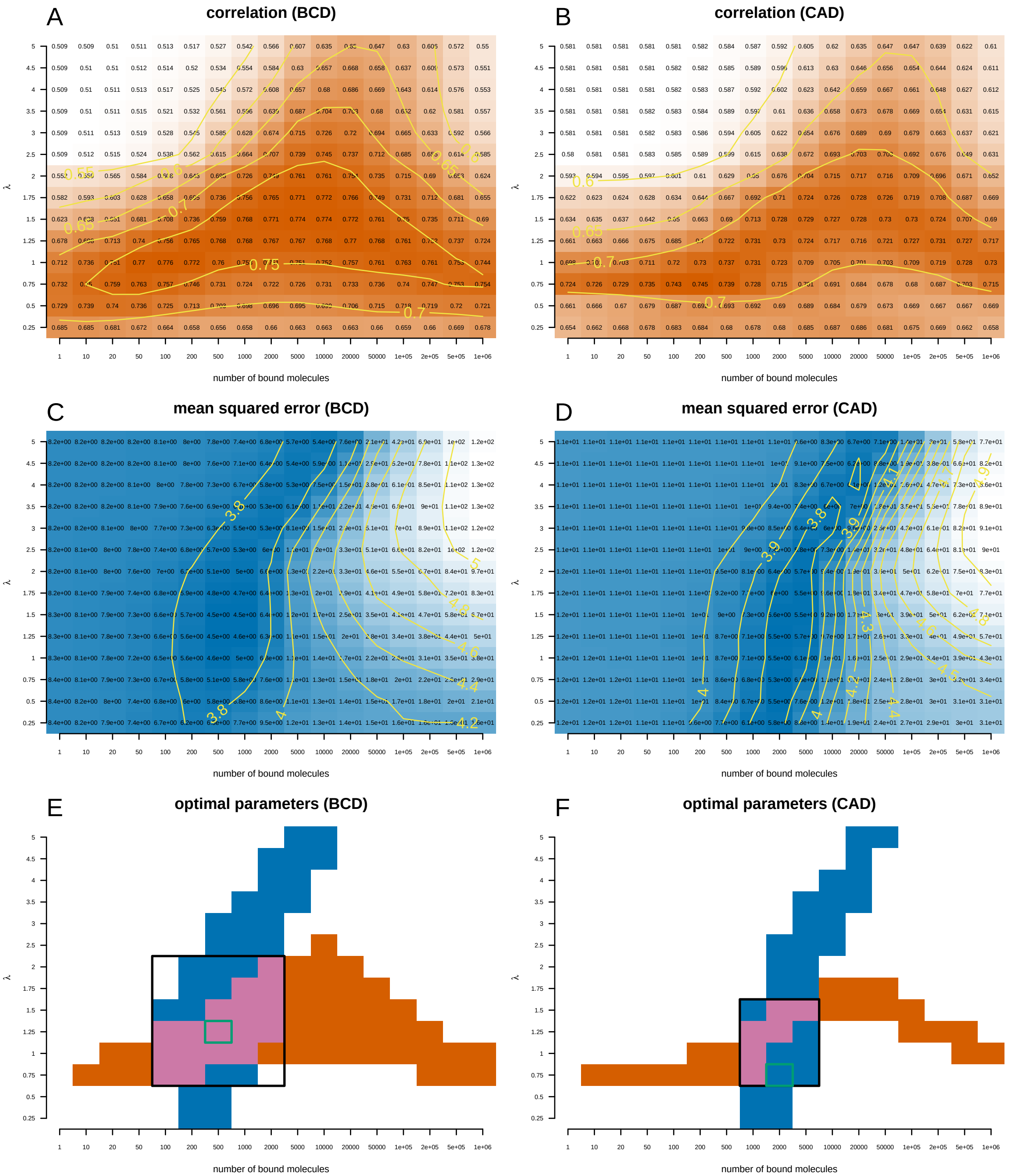

Figure S13: *Estimating the Bicoid and Caudal ChIP-seq profiles when assuming non-binary accessibility data*. We plotted heatmaps for the correlation (A) and (B) and mean squared error (C) and (D) between the analytical model and the ChIP-seq profile of Bicoid (A, C) and Caudal (B, D). The analytical model includes non-binary DNA accessibility data, by using equation (S9) to compute the probability that a site is accessible. We computed these values for different sets of parameters: $N \in [1, 10^6]$ and $\lambda \in [0.25, 5]$. Colour code as above. We considered only the sites that have a PWM score higher than 70% of the difference between the lowest and the highest score. (E, F) We plotted the regions where the mean squared error is in the lower 12% of the range of values (blue) and the correlation is the higher 12% of the range of values (orange). With green rectangle we marked the optimal set of parameters in terms of mean squared error and with a black rectangle the intersection of the parameters for which the two regions intersect.

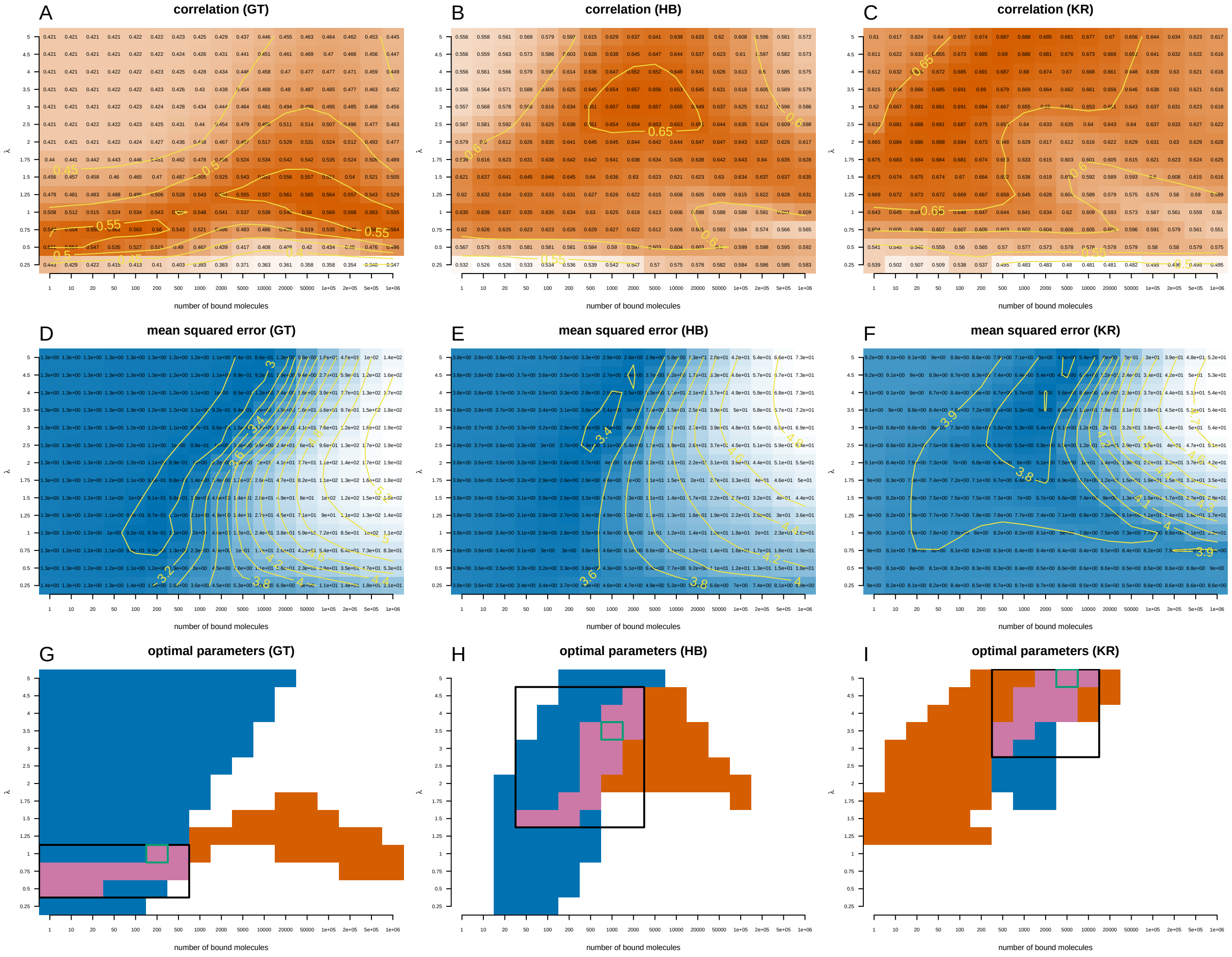

Figure S14: *Estimating the Giant, Hunchback and Kruppel caudal ChIP-seq profiles when assuming non-binary accessibility data.* We plotted heatmaps for the correlation $(A-C)$ and mean squared error $(D-F)$ between the analytical model and the ChIP-seq profile of Giant (A, D), Hunchback (B, E) and Kruppel (C, F). The analytical model includes non-binary DNA accessibility data, by using equation (S9) to compute the probability that a site is accessible. We computed these values for different sets of parameters: $N \in [1, 10^6]$ and $\lambda \in [0.25, 5]$. Colour code as above. PWM filtering as before. $(G-I)$ We plotted the regions where the mean squared error is in the lower 12% of the range of values (blue) and the correlation is the higher 12% of the range of values (orange). With green rectangle we marked the optimal set of parameters in terms of mean squared error and with a black rectangle the intersection of the parameters for which the two regions intersect.

S4.5 Using the JASPAR PWMs

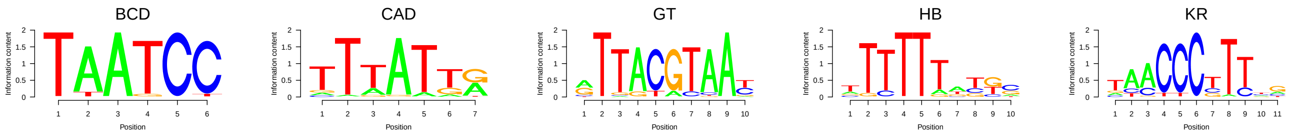

Figure S15: *PWMs for the five TFs from the JASPAR database.* The graph plots the sequence logos for the following TFs: (*i*) Bicoid, (*ii*) Caudal, (*iii*) Giant, (*iv*) Hunchback and (*v*) Kruppel [18]. When computing the PWMs we used a pseudo count of 1. The information content for the five motifs is: (*i*) $I_{BCD} = 11.0$, (*ii*) $I_{CAD} = 8.9$, (*iii*) $I_{GT} = 14.0$, (*iv*) $I_{HB} = 10.4$ and (*v*) $I_{KR} = 11.7$.

	N	λ	MSE	ρ
BCD	5000	1.00	4.06 (4.06)	0.74 (0.75)
CAD	10000	1.25	6.23 (6.23)	0.64 (0.67)
GT	10000	4.50	0.86 (0.86)	0.54 (0.56)
HB	2000	1.00	2.56 (2.56)	0.68 (0.68)
KR	5000	2.00	4.73 (4.73)	0.67 (0.69)

Table S6: *Set of parameters that minimises the difference between the ChIP-seq profiles when using the binding motif from JASPAR database* [18]; see Figure S15. Our model assumes binary DNA accessibility data (the accessibility of any site can be either 0 or 1 depending on whether the site is accessible or not). We also listed the values for the mean squared error (MSE) and correlation (ρ). The values in the parentheses represent the minimum mean squared error and the maximum correlation. We considered only the sites that have a PWM score higher than 70% of the distance between the lowest and the highest score.

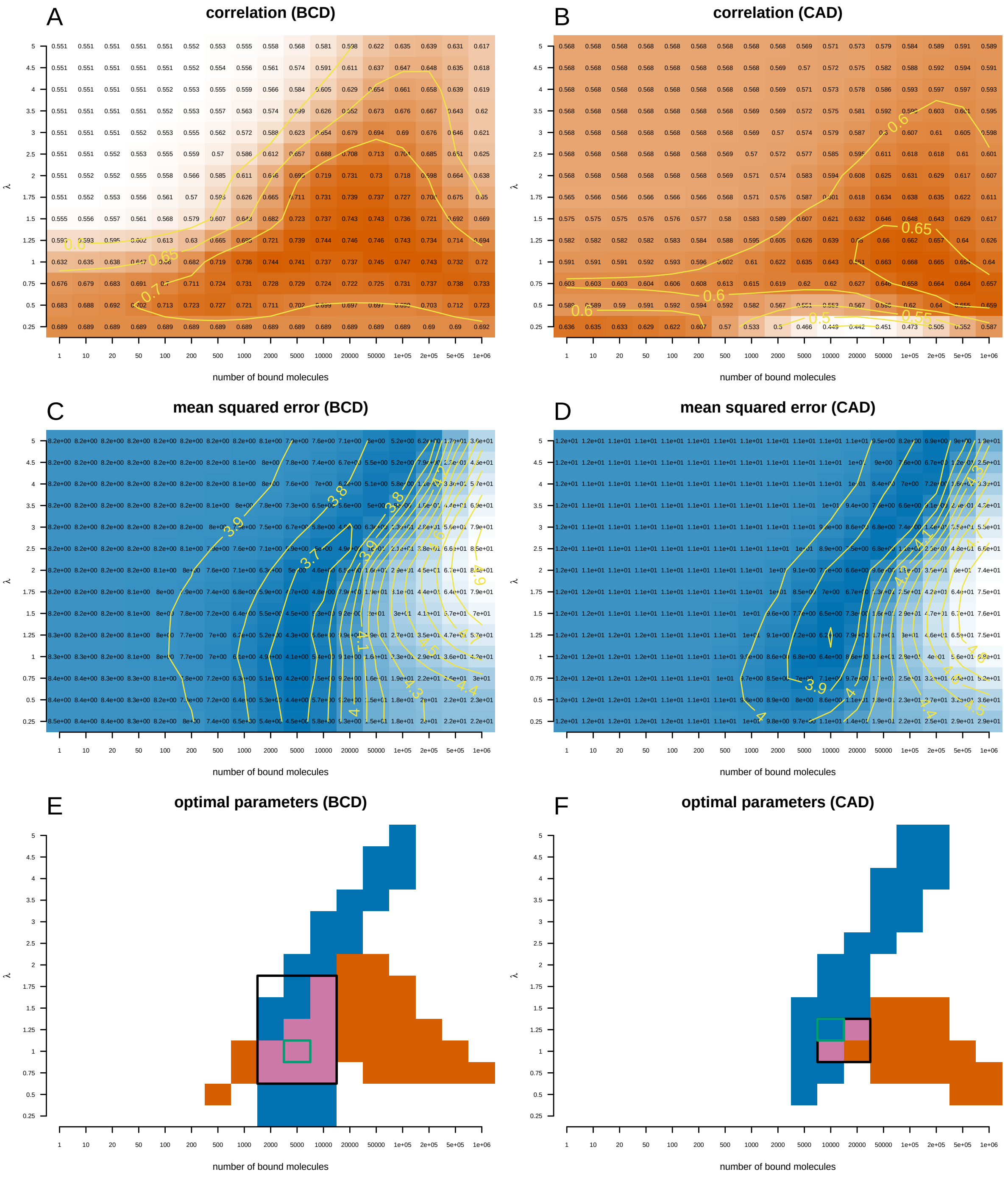

Figure S16: *Estimating the Bicoid and Caudal ChIP-seq profiles when using the binding motif from JASPAR database*; see Figure S15. We plotted heatmaps for the correlation (A) and (B) and mean squared error (C) and (D) between the analytical model and the ChIP-seq profile of Bicoid (A, C) and Caudal (B, D). The analytical model includes binary DNA accessibility data (the accessibility of any site can be either 0 or 1 depending on whether the site is accessible or not). We computed these values for different sets of parameters: $N \in [1, 10^6]$ and $\lambda \in [0.25, 5]$. Colour code as above. We considered only the sites that have a PWM score higher than 70% of the difference between the lowest and the highest score. (E, F) We plotted the regions where the mean squared error is in the lower 12% of the range of values (blue) and the correlation is the higher 12% of the range of values (orange). With green rectangle we marked the optimal set of parameters in terms of mean squared error and with a black rectangle the intersection of the parameters for which the two regions intersect.

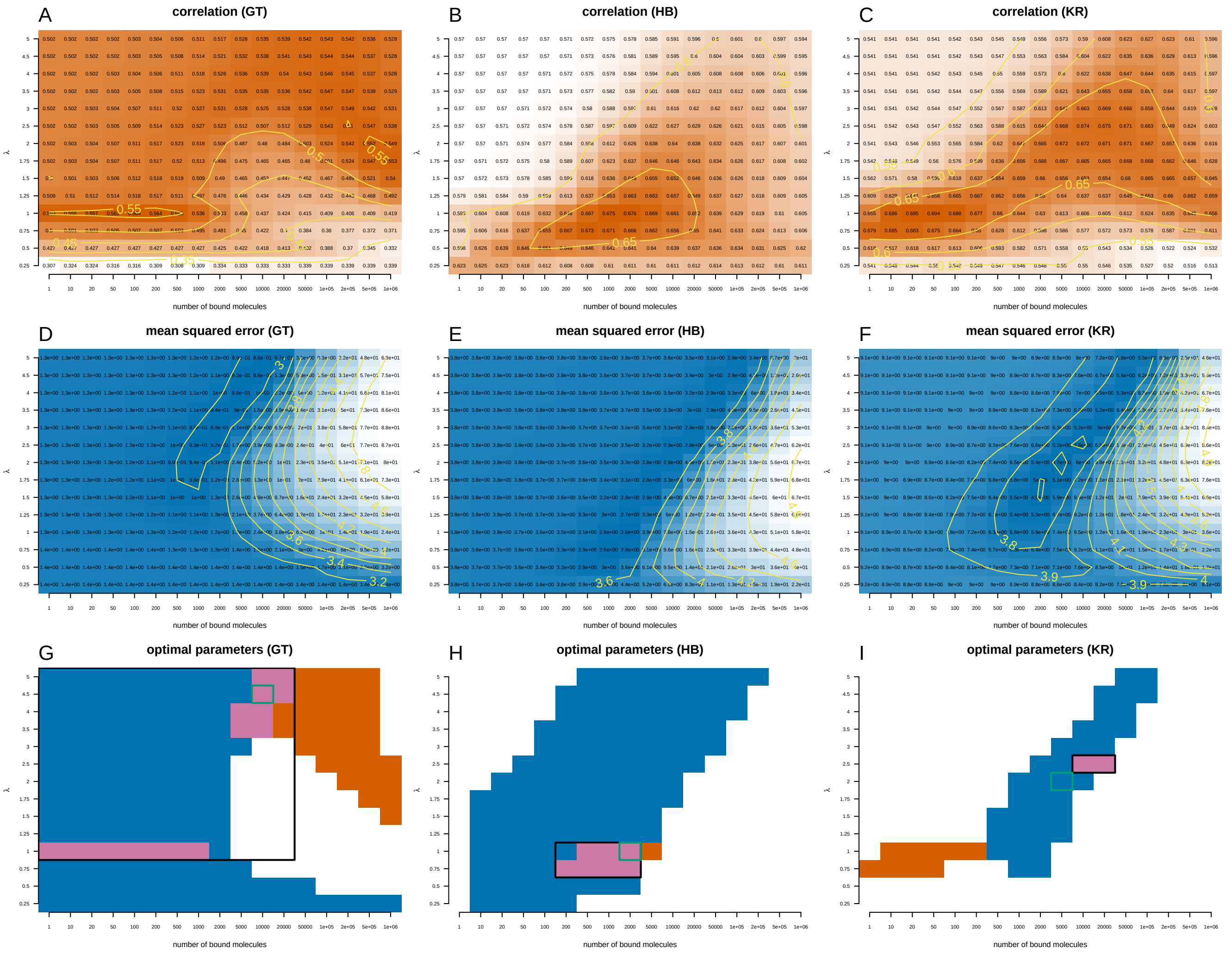

Figure S17: *Estimating the Giant, Hunchback, Kruppel and Caudal ChIP-seq profiles when using the binding motif from JASPAR database*; see Figure S15. We plotted heatmaps for the correlation $(A-C)$ and mean squared error $(D-F)$ between the analytical model and the ChIP-seq profile of Giant (A, D), Hunchback (B, E) and Kruppel (C, F). The analytical model includes binary DNA accessibility data (the accessibility of any site can be either 0 or 1 depending on whether the site is accessible or not). We computed these values for different sets of parameters: $N \in [1, 10^6]$ and $\lambda \in [0.25, 5]$. Colour code as above. PWM filtering as above. $(G-I)$ We plotted the regions where the mean squared error is in the lower 12% of the range of values (blue) and the correlation is the higher 12% of the range of values (orange). With green rectangle we marked the optimal set of parameters in terms of mean squared error and with a black rectangle the intersection of the parameters for which the two regions intersect.

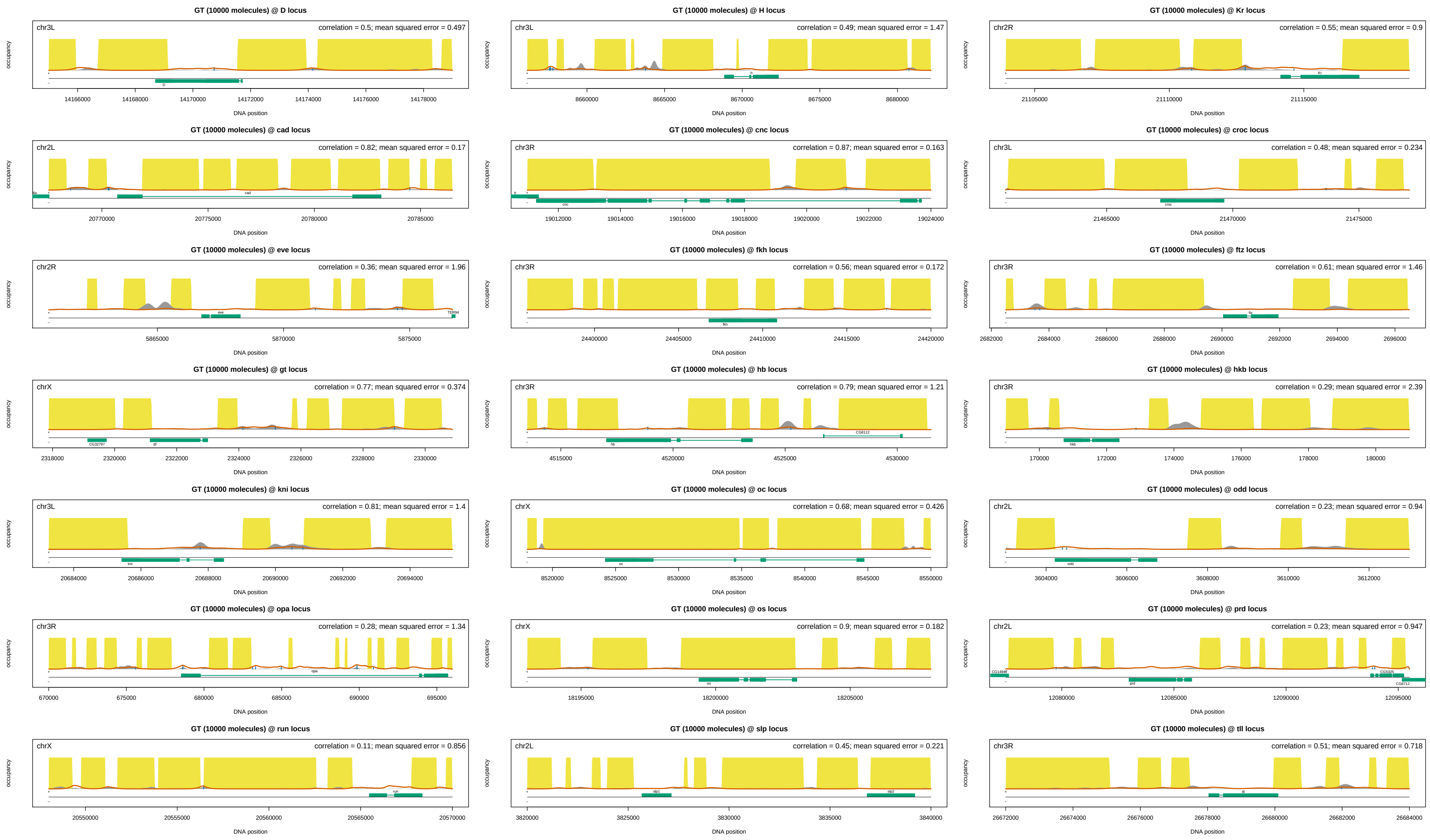

Figure S18: *Binding profiles for Giant at all 21 loci using the JASPAR database PWM.* The grey shading represents a ChIP-seq profile, the red line represents the prediction of the analytical model, the yellow shading represents the inaccessible DNA and the vertical blue lines represent the percentage of occupancy of the site (we only displayed sites with an occupancy higher than 5%). We considered the optimal set of parameters for hunchback (10000 *molecules* and $\lambda = 4.50$).

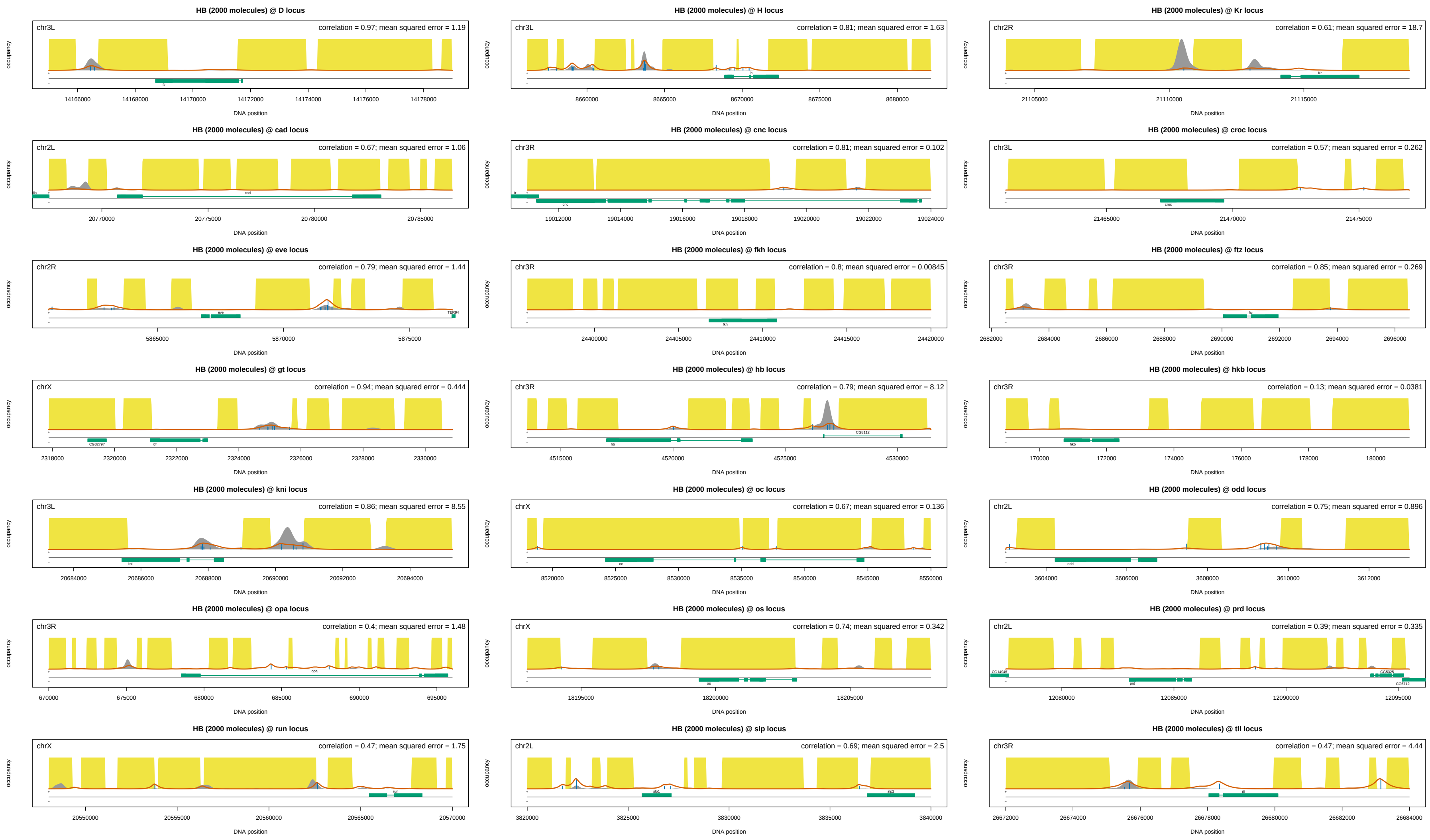

Figure S19: *Binding profiles for Hunchback at all 21 loci using the JASPAR database PWM.* The grey shading represents a ChIP-seq profile, the red line represents the prediction of the analytical model, the yellow shading represents the inaccessible DNA and the vertical blue lines represent the percentage of occupancy of the site (we only displayed sites with an occupancy higher than 5%). We considered the optimal set of parameters for hunchback (2000 *molecules* and $\lambda = 1.00$).

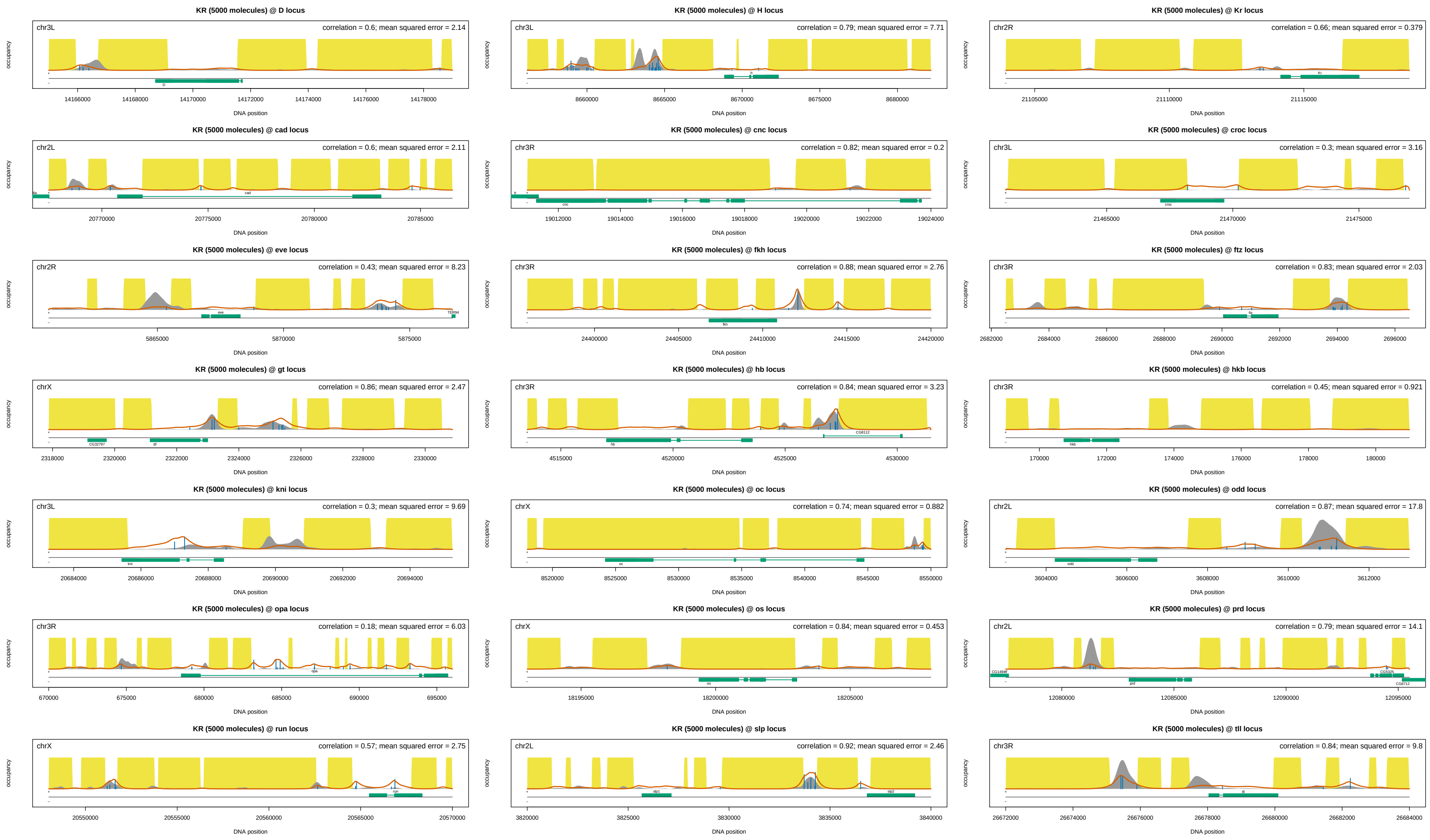

Figure S20: *Binding profiles for Kruppel at all 21 loci using the JASPAR database PWM.* The grey shading represents a ChIP-seq profile, the red line represents the prediction of the analytical model, the yellow shading represents the inaccessible DNA and the vertical blue lines represent the percentage of occupancy of the site (we only displayed sites with an occupancy higher than 5%). We considered the optimal set of parameters for Kruppel (5000 *molecules* and $\lambda = 2.00$).

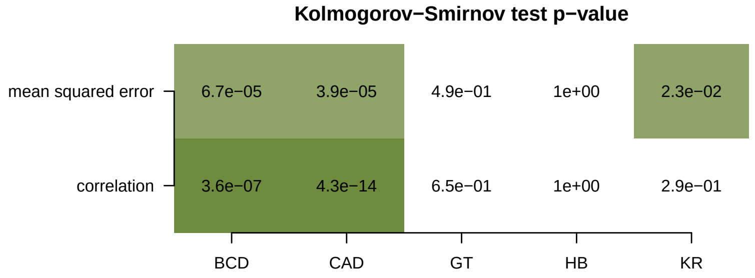

Figure S21: *The p-value of the KolmogorovSmirnov test between the two subsets of thresholds.* We performed a Kolmogorov-Smirnov test between the case when we selected the regions with a mean ChIP-seq signal higher than the background ($> B$) and the case when we selected the regions with a mean ChIP-seq signal higher than half the background ($> 0.5 \cdot B$). We considered both the mean squared error and correlation coefficient for each of the five TFs.

S4.6 Genome-wide analysis

	BCD	CAD	GT	HB	KR
B	0.03	0.03	0.04	0.04	0.04
M	1.85	1.63	4.97	16.23	5.23

Table S7: *Mean and maximum of the ChIP-seq signal for the five TFs.*

	BCD	CAD	GT	HB	KR
1.0	35	167	1	2	8
0.5	812	1984	32	6	83

Table S8: *The number of DNA segments with a ChIP-seq signal higher than the threshold.* We considered two thresholds $K = 1.0$ and $K = 0.5$. The mean ChIP-seq signal of the 20 Kbp segment needs to be higher than $K \times B$.

TF	cell type	% of bound TF	experiment type	type of binding	reference
C/EBP	HeLa	35.5%	FRAP	specific	[19]
NF1	HeLa	58.1%	FRAP	specific	[19]
Jun	HeLa	75.2%	FRAP	specific	[19]
Fos	HeLa	54.2%	FRAP	specific	[19]
Myc	HeLa	54.1%	FRAP	specific	[19]
Max	HeLa	34.5%	FRAP	specific	[19]
Max	3134	16.0%	FRAP	specific	[20]
Mad	HeLa	21.2%	FRAP	specific	[19]
FBP	HeLa	99.7%	FRAP	specific	[19]
XBP	HeLa	18.6%	FRAP	specific	[19]
BRD4	HeLa	62.4%	FRAP	specific	[19]
p53	H1299	7.0%	FRAP/FCS/SMT	specific	[21]
p53	H1299	2.5%	SMT	specific	[22]
GR	MCF-7	12.0%	RLSM	specific	[23]
GR*	MCF-7	37.0%	RLSM	specific	[23]
GR	3134	3.5%	SMT	specific	[22]
STAT1*	HeLa	34.0%	SMT	specific	[24]
HSF1	U87	30.0%	FCS	specific	[25]
HSF1*	U87	45.0%	FCS	specific	[25]
Sox2	ES	15.1%	2D SMT	specific	[26]
Sox2 (with Oct4*)	NIH 3T3	16.3%	2D SMT	specific	[26]
Sox2 (with Oct4)	NIH 3T3	16.9%	2D SMT	specific	[26]
Oct4	ES	14.4%	2D SMT	specific	[26]
Oct4 (with Sox2*)	NIH 3T3	18.4%	2D SMT	specific	[26]
Oct4 (with Sox2)	NIH 3T3	10.7%	2D SMT	specific	[26]

Table S9: *The fraction of bound molecules.* This is a (non exhaustive) list of the percentage of bound molecules of several TFs to the DNA as determined experimentally in previous works. The following methods were used: Fluorescence Recovery After Photobleaching (FRAP), Fluorescence Correlation Spectroscopy (FCS), Single Molecule Tracking (SMT) and Reflected Light-Sheet Microscope (RLSM). The $*$ superscript indicates the TF in induced state. Note that if the same group published different measurements for the same TF, we only selected the latest value. In addition, if the data was fitted to a model assuming two modes of binding, we report only the faction of bound molecules in the slower moving component, which estimates the fraction of specifically bound TF. The distribution of the percentage of bound TFs is plotted in Figure S22.

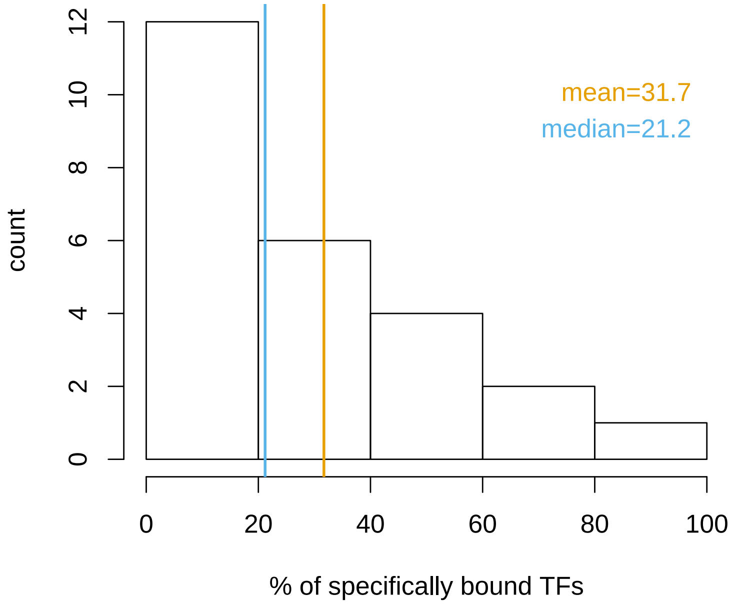

Figure S22: *The distribution of the percentage of specifically bound molecules for different TFs.* We plotted the data from Table S9.

	min abundance	max abundance	min specifically bound	max specifically bound
BCD	10000	30000	7%	20%
CAD	12000	37000	27%	81%
GT	23000	70000	1%	4%
HB	11000	34000	6%	18%
KR	37000	110000	18%	54%

Table S10: *The TF abundance in the nucleus and the percentage of specifically bound TF.* In the first two columns, we list the number of molecules that are in the nucleus for the five TFs. For our estimates for Bicoid nuclear abundance, see the Discussion section in the main text. In [27], the authors reanalysed the FlyEx data [28] and proposed a lower limit for the nuclear abundance of the five TFs, but the proposed values are underestimates of the real values. For the last four TFs (Caudal, Giant, Hunchback and Kruppel), we considered the nuclear abundances of the four TFs relative to Bicoid nuclear abundance, as estimated in [27] using the Poisson method, and then we multiplied these relative abundances with our estimates for the Bicoid nuclear abundance. In the last two columns we list the percentage of specifically bound TFs, based on the estimations of our method (Table 2 in the main text) and the values in the first two columns.

References

[1] Ulrich Gerland, J. David Moroz, and Terence Hwa. Physical constraints and functional characteristics of transcription factor-DNA interactions. *PNAS*, 99(19):12015–12020, 2002.

[2] Otto G. Berg and Peter H. von Hippel. Selection of DNA binding sites by regulatory proteins statistical-mechanical theory and application to operators and promoters. *Journal of Molecular Biology*, 193(4):723–750, 1987.

[3] Helge G. Roider, Aditi Kanhere, Thomas Manke, and Martin Vingron. Predicting transcription factor affinities to DNA from a biophysical model. *Bioinformatics*, 23(2):134–141, 2007.

[4] Nicolae Radu Zabet and Boris Adryan. A comprehensive computational model of facilitated diffusion in prokaryotes. *Bioinformatics*, 28(11):1517–1524, 2012.

[5] Nicolae Radu Zabet and Boris Adryan. GRiP: a computational tool to simulate transcription factor binding in prokaryotes. *Bioinformatics*, 28(9):1287–1289, 2012.

[6] Nicolae Radu Zabet. System size reduction in stochastic simulations of the facilitated diffusion mechanism. *BMC Systems Biology*, 6(1):121, 2012.

[7] Nicolae Radu Zabet and Boris Adryan. Computational models for large-scale simulations of facilitated diffusion. *Molecular BioSystems*, 8(11):2815–2827, 2012.

[8] Nicolae Radu Zabet, Robert Foy, and Boris Adryan. The influence of transcription factor competition on the relationship between occupancy and affinity. *PLoS ONE*, 8(9):e73714, 2013.

[9] Gary K. Ackers, Alexander D. Johnson, and Madeline A. Shea. Quantitative model for gene regulation by lambda phage repressor. *PNAS*, 79:1129–1133, 1982.

[10] Lacramioara Bintu, Nicolas E. Buchler, Hernan G. Garcia, Ulrich Gerland, Terence Hwa, Jane Kondev, and Rob Phillips. Transcriptional regulation by the numbers: models. *Current Opinion in Genetics & Development*, 15(2):116–124, 2005.

[11] Lacramioara Bintu, Nicolas E. Buchler, Hernan G. Garcia, Ulrich Gerland, Terence Hwa, Jane Kondev, and Rob Phillips. Transcriptional regulation by the numbers: applications. *Current Opinion in Genetics & Development*, 15(2):125–135, 2005.

[12] Dominique Chu, Nicolae Radu Zabet, and Boris Mitavskiy. Models of transcription factor binding: Sensitivity of activation functions to model assumptions. *Journal of Theoretical Biology*, 257(3):419–429, 2009.

[13] Jovan Simicevic, Adrien W Schmid, Paola A Gilardoni, Benjamin Zoller, Sunil K Raghav, Irina Krier, Carine Gubelmann, Frederique Lisacek, Felix Naef, Marc Moniatte, and Bart Deplancke. Absolute quantification of transcription factors during cellular differentiation using multiplexed targeted proteomics. *Nature Methods*, 10(6):570–576, 2013.

[14] M Sheinman and Y Kafri. How does the DNA sequence affect the Hill curve of transcriptional response? *Physical Biology*, 9(5):056006, 2012.

[15] Tommy Kaplan, Xiao-Yong Li, Peter J. Sabo, Sean Thomas, John A. Stamatoyannopoulos, Mark D. Biggin, and Michael B. Eisen. Quantitative models of the mechanisms that control genome-wide patterns of transcription factor binding during early *Drosophila* development. *PLoS Genetics*, 7(2):e1001290, 2011.

[16] Petter Hammar, Prune Leroy, Anel Mahmutovic, Erik G. Marklund, Otto G. Berg, and Johan Elf. The lac repressor displays facilitated diffusion in living cells. *Science*, 336(6088):1595–1598, 2012.

[17] Jeroen S. van Zon, Marco J. Morelli, Sorin Tanase-Nicola, and Pieter Rein ten Wolde. Diffusion of transcription factors can drastically enhance the noise in gene expression. *Biophysical Journal*, 91(12):4350–4367, 2006.

[18] Elodie Portales-Casamar, Supat Thongjuea, Andrew T. Kwon, David Arenillas, Xiaobei Zhao, Eivind Valen, Dimas Yusuf, Boris Lenhard, Wyeth W. Wasserman, and Albin Sandelin. JASPAR 2010: the greatly expanded open-access database of transcription factor binding profiles. *Nucleic Acids Research*, 38(suppl 1):D105–D110, 2010.

[19] Robert D. Phair, Paola Scaffidi, Cem Elbi, Jaromra Vecerov, Anup Dey, Keiko Ozato, David T. Brown, Gordon Hager, Michael Bustin, and Tom Misteli. Global nature of dynamic protein-chromatin interactions in vivo: Three-dimensional genome scanning and dynamic interaction networks of chromatin proteins. *Molecular and Cellular Biology*, 24(14):6393–6402, 2004.

[20] Florian Mueller, Paul Wach, and James G. McNally. Evidence for a common mode of transcription factor interaction with chromatin as revealed by improved quantitative fluorescence recovery after photobleaching. *Biophysical Journal*, 94(8):3323–3339, 2008.

[21] Davide Mazza, Alice Abernathy, Nicole Golob, Tatsuya Morisaki, and James G. McNally. A benchmark for chromatin binding measurements in live cells. *Nucleic Acids Research*, 40(15):e119, 2012.

[22] Tatsuya Morisaki, Waltraud G. Muller, Nicole Golob, Davide Mazza, and James G. McNally. Single-molecule analysis of transcription factor binding at transcription sites in live cells. *Nature Communications*, 5:4456, 2014.

[23] J Christof M Gebhardt, David M Suter, Rahul Roy, Ziqing W Zhao, Alec R Chapman, Srinjan Basu, Tom Maniatis, and X Sunney Xie. Single-molecule imaging of transcription factor binding to DNA in live mammalian cells. *Nature Methods*, 10(5):421–426, 2013.

[24] Jasmin Speil, Eugen Baumgart, Jan-Peter Siebrasse, Roman Veith, Uwe Vinkemeier, and Ulrich Kubitscheck. Activated STAT1 transcription factors conduct distinct saltatory movements in the cell nucleus. *Biophysical Journal*, 101(11):2592–2600, 2011.

[25] Meike Kloster-Landsberg, Gaetan Herbomel, Irne Wang, Jacques Derouard, Claire Vourch, Yves Usson, Catherine Souchier, and Antoine Delon. Cellular response to heat shock studied by multiconfocal fluorescence correlation spectroscopy. *Biophysical Journal*, 103(6):1110–1119, 2011.

[26] Jiji Chen, Zhengjian Zhang, Li Li, Bi-Chang Chen, Andrey Revyakin, Bassam Hajj, Wesley Legant, Maxime Dahan, Timothée Lionnet, Eric Betzig, Robert Tjian, and Zhe Liu. Single-molecule dynamics of enhanceosome assembly in embryonic stem cells. *Cell*, 156(6):1274–1285, 2014.

[27] Lee Zamparo and Theodore J. Perkins. Statistical lower bounds on protein copy number from fluorescence expression images. *Bioinformatics*, 25(20):2670–2676, 2009.

[28] Andrei Pisarev, Ekaterina Poustelnikova, Maria Samsonova, and John Reinitz. FlyEx, the quantitative atlas on segmentation gene expression at cellular resolution. *Nucleic Acids Research*, 37(suppl 1):D560–D566, 2009.
